# Functional connectivity profile of the human inferior frontal junction: involvement in a cognitive control network

**DOI:** 10.1186/1471-2202-13-119

**Published:** 2012-10-03

**Authors:** Benedikt Sundermann, Bettina Pfleiderer

**Affiliations:** 1 Department of Clinical Radiology, University Hospital Münster, Albert-Schweitzer-Campus 1, Gebäude A1, 48149, Münster, Germany

**Keywords:** Cognition, Prefrontal cortex, Magnetic resonance imaging, MACM, Meta-analysis, Resting state

## Abstract

**Background:**

The human inferior frontal junction area (IFJ) is critically involved in three main component processes of cognitive control (working memory, task switching and inhibitory control). As it overlaps with several areas in established anatomical labeling schemes, it is considered to be underreported as a functionally distinct location in the neuroimaging literature. While recent studies explicitly focused on the IFJ's anatomical organization and functional role as a single brain area, it is usually not explicitly denominated in studies on cognitive networks. However based on few analyses in small datasets constrained by specific a priori assumptions on its functional specialization, the IFJ has been postulated to be part of a cognitive control network. Goal of this meta-analysis was to establish the IFJ’s connectivity profile on a high formal level of evidence by aggregating published implicit knowledge about its co-activations. We applied meta-analytical connectivity modeling (MACM) based on the activation likelihood estimation (ALE) method without specific assumptions regarding functional specialization on 180 (reporting left IFJ activity) and 131 (right IFJ) published functional neuroimaging experiments derived from the BrainMap database. This method is based on coordinates in stereotaxic space, not on anatomical descriptors.

**Results:**

The IFJ is significantly co-activated with areas in the dorsolateral and ventrolateral prefrontal cortex, anterior insula, medial frontal gyrus / pre-SMA, posterior parietal cortex, occipitotemporal junction / cerebellum, thalamus and putamen as well as language and motor areas. Results are corroborated by an independent resting-state fMRI analysis.

**Conclusions:**

These results support the assumption that the IFJ is part of a previously described cognitive control network. They also highlight the involvement of subcortical structures in this system. A direct line is drawn from works on the functional significance of brain activity located at the IFJ and its anatomical definition to published results related to distributed cognitive brain systems. The IFJ is therefore introduced as a convenient starting point to investigate the cognitive control network in further studies.

## Background

The human inferior frontal junction area (IFJ) located at the junction of the inferior frontal sulcus and the inferior precentral sulcus had been largely neglected as a distinct region involved in cognitive control processes. Yet recent work explicitly addressing this brain area has attributed a major role to the IFJ related to three main component processes (task switching, inhibitory control and working memory) [1-8]. In an FDG-PET study hypometabolism in the IFJ was associated with cognitive decline in early dementia suggesting a potential clinical relevance of IFJ functioning [5]. An involvement of the IFJ in a network associated with cognitive control has been suggested mainly based on a single highly hypothesis-driven combined task- and resting-state-fMRI study. This network also involves the anterior cingulate cortex/pre-supplementary motor area (ACC/pre-SMA), dorsolateral prefrontal cortex (DLPFC), anterior insular cortex, dorsal pre-motor cortex, and posterior parietal cortex (PPC) [9]. Though there is a larger body of neuroimaging literature on fronto-parietal networks associated with cognitive functions (e.g. [10,11], see Discussion), to our knowledge there is no further work explicitly denominating the IFJ as one of their constituents.

Peak coordinates from published human functional magnetic resonance imaging (fMRI) studies accumulated in the BrainMap database have previously been integrated in order to delineate the functional connectivity of designated brain regions based on their co-activation profiles using the activation likelihood estimation (ALE) method [12-14]: Activation coordinates reported together with peaks within a defined seed region are retrieved from the database. Gaussian probability distributions are then modeled centered at these coordinates based on spatial variability estimates. Then these distributed activations are analyzed for where they converge in random-effects-analyses, weighted by the number of subjects in the studies included [12,15]. Such Metanalytic Connectivity Modelling (MACM) has been validated against non-human primate anatomical connectivity [16] and human resting-state functional connectivity (rsFC) modeling [13] showing substantial overlap. There is additional evidence that large BrainMap datasets contain information similar to rsFC from calculations based on independent component analyses (ICA) [10]. A similar coactivation approach has been used to build a voxel-wise functional connectivity map of the human brain using the BrainMap database in June 2006 with 825 articles available then [17].

Goal of this meta-analysis was to aggregate implicit knowledge by means of coordinate representation (independent from anatomical descriptors) to characterize the functional connectivity profile of the IFJ on the basis of more than a decade of neuroimaging studies. These included a variety of studies investigating cognitive control yet there were no a priori constraints regarding the functional specialization of the IFJ. Do these aggregated data support the hypothesis of the involvement in the cognitive control network as proposed by Cole et al. [9]? Are there additional regions associated with IFJ activations not recognized by many individual studies but significantly involved across a large number of fMRI sessions? How do potential findings relate to earlier work based on different methodology (human structural connectivity based on diffusion MRI, human resting-state and task-based fMRI and animal studies) regarding the role of the IFJ in the functional and structural organization of the human brain? To what extent is the IFJ-connectivity lateralized? Can meta-analytic results be confirmed by resting-state fMRI [18] using an analogous seed-definition?

## Methods

### Activation likelihood estimation meta-analysis

IFJ coordinates were adapted from a review article [1] aggregating peak locations from different functional imaging studies, and task-based meta-analyses showing IFJ-activations and reporting an approximation of the IFJ location as a functionally defined brain area. Instead, relying on an atlas-based anatomical definition would have been difficult as the IFJ has been reported to overlap with different cytoarchitectonically defined areas (mainly Brodmann areas 6, 9 and 44). Cuboidal seeds were defined by adding spatial extent along the x-axis to their two-dimensional definition: left IFJ from (-52, 1, 27) to (-42, 10, 40) and right IFJ from (42, 1, 27) to (52, 10, 40) with (x, y, z) representing coordinates in the Talairach coordinate system [19]. All coordinates reported in the meta-analysis refer to Talairach space, which is the space used by the BrainMap database.

Data were retrieved from the BrainMap database using Sleuth (Version 1.2) [20,21] on 30 June 2011 containing results of 2,114 articles in total at that time [22]. Whole-brain activation coordinates from those fMRI sessions which revealed IFJ activations within the seeds were extracted for the left and right IFJ separately. The query was additionally restricted to right-handed healthy subjects and the context of normal mapping according to the studies’ BrainMap records.

Regarding the left IFJ we identified 180 experiments/contrasts comprising 2,274 subjects in 139 articles (from 730 single experiments in 2,434 participants in these studies in total). Consequently 2,764 of 8,301 locations reported in these papers were included in further analyses. For the right IFJ 131 experiments in 1,767 subjects reported in 111 articles (from 574 experiments in 1962 participants in these studies) matched these criteria. Thus 2,336 of 6,922 locations shown in these papers were included. A complete list of these articles is provided in the supplementary online material.

The ALE random effects meta-analysis was conducted using the revised algorithm by Eickhoff et. al. in GingerALE (Version 2.0) [15,23]. After excluding locations potentially outside the brain by masking coordinates using the conservative standard mask in GingerALE (dimensions 80 x 96 x 70 mm) 2,727 (left IFJ) and 2,293 activation foci (right IFJ) remained. Study specific smoothing using a Gaussian kernel was applied (left IFJ: median full width at half maximum = 9.66, range 8.71 to 11.37; right IFJ: median full with at half maximum = 9.66, range 8.67 to 11.37). For resulting co-activations with the IFJ a threshold of p = 0.001 corrected for multiple comparisons using false-discovery rate (FDR) was applied at voxel-level. Anatomical labels are automatically assigned in GingerALE.

Visualizations were created using Mango (Version 2.3.2) [24] and an anatomical template in Talairach space [25].

### Resting-state functional connectivity analysis

In order to confirm our meta-analytic results using a different approach to functional connectivity, we additionally performed a seed-based analysis of intrinsic fluctuations in a presumably independent sample.

We therefore retrieved an anonymized resting-state fMRI dataset comprising 198 individuals (123 females and 75 males aged 18 to 30 years, mean age: 21.03 ± 2.31 years). For each participant 119 volumes of 47 slices had been acquired with TR = 3 s using a 3 Tesla MRI-scanner (resulting total acquisition time 5 min 57 s). The dataset is publicly available from the 1000 Functional Connectomes Project (FCP) [26] and data were provided by R. L. Buckner, Howard Hughes Medical Institute, Cambridge, MA. Acquisition and submission of the data was approved by the contributor’s ethics committee. The institutional review boards of NYU Langone Medical Center and New Jersey Medical School approved the receipt and dissemination of the data through the FCP [27].

As a prerequisite for further analyses, center coordinates of the meta-analytic seed as described above were converted to MNI space using the Lancaster transform [28] as implemented in GingerALE [29]: ±52, 11, 31 (seeds 1 and 2). In order to exclude a substantial biasing influence of our seed coordinate selection strategy we also conducted an analogous analysis using more medial mean coordinates from a study on interindividual variability of the IFJ’s location [2]: -41, 7, 31 and 47, 7, 29 (seeds 3 and 4). Spherical ROIs with a radius of 15 mm in MNI spaced were constructed and used as seeds in further analyses.

There is a range of widely accepted preprocessing steps in analyses of rs-fc data [30]. The following raw data modification steps were guided by these principles: The first 6 EPI-volumes were discarded in order to allow for T1-equilibration. Using SPM8 [31] based on MATLAB (R2010a, ver. 7.10.0.499, The MathWorks Inc., Natick, MA) images were motion corrected, normalized to a template in MNI space, resampled to a voxel-size of 2 x 2 x 2 mm^3^ and smoothed using a Gaussian kernel (FWHM = 6 mm).

Resulting images were subjected to a conventional seed-based functional connectivity analysis based on Pearson linear correlation using REST (version 1.7) [32,33], a MATLAB Toolbox: A temporal band-pass filter (0.01 to 0.08 Hz) was applied to restrict the analysis to the typical frequency-band of spontaneous signal fluctuations and linear trends were removed. A template was used for masking voxels typically outside the brain. Signal time courses obtained from the ventricles and white matter as operationalized by corresponding templates, global signal and motion correction parameters were included as covariates in the partial correlation model. Correlation coefficients of signal co-fluctuations with the left IFJ were calculated for every remaining voxel and Z-scored. This resulted in one unthresholded correlation map for each individual subject for each IFJ seed. These preprocessing steps based on SPM8 and REST were accomplished using the Data Processing Assistant for Resting-State fMRI (DPARSF, version 2.1) [34].

Resulting correlation maps were subjected to a one-sample t-test in SPM8. Results were thresholded at p < 0.001, corrected for multiple comparisons using family-wise error-rate (FWE). For comparison with the MACM results, peak-coordinates of rs-fc were transformed back to Talairach space. Local maxima of the correlation maps (at least 8 mm apart) were compared to meta-analytic results and assigned to brain areas as named in the results and discussion section of the meta-analysis. Additional brain regions were labeled based on the Talairach atlas [35,36].

## Results

### Meta-analytic results

**Figure 1:**
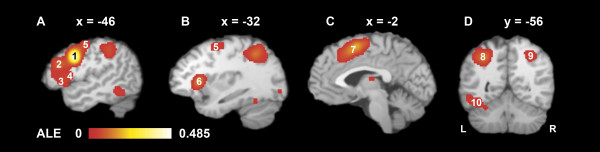
**Activation likelihood of cortical areas associated with left IFJ activations.** (p < 0.001, FDR corrected): **a**) and **b**) subregions of the frontal/insular cluster of the left hemisphere (1 inferior frontal gyrus / IFJ, 2 middle frontal gyrus, 3 inferior frontal gyrus, 4 precentral gyrus, 5 precentral gyrus, 6 insula) **c**) postero-medial frontal cortex (7 left medial frontal gyrus) **d**) posterior regions (8 left superior / inferior parietal lobule, 9 right inferior parietal lobule, 10 left fusiform gyrus / inferior temporal gyrus / cerebellum.

The IFJ was associated bilaterally with a set of frontal and insular activations, mainly contained as separate peaks in an expansive cluster spanning anatomically distinct regions. It is exemplarily depicted in Figure 1a) and b) in terms of left hemispherical co-activations with the left seed. Peak activations within the IFJ seeds and corresponding contralateral activations were anatomically labeled inferior frontal gyrus, Brodmann area (BA) 9 or precentral gyrus (BA 6) based on the Talairach atlas. The fronto-insular co-activations comprised an area of the dorso-lateral prefrontal cortex (DLPFC) on the middle frontal gyrus, confluent ventro-lateral prefrontal areas with neighboring peak coordinates in the inferior frontal gyrus and precentral gyrus (including BA 44 in the left hemisphere), a posterior dorsal area predominantly in the precentral gyrus (BA 4 and 6) and parts of the anterior insula. A cluster in the posterior fronto-median cortex was evident independent from the other frontal locations; see Figure 1c).

**Figure 2:**
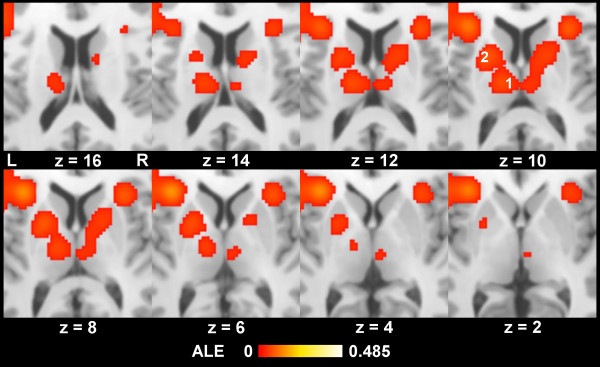
**Subcortical activation likelihood associated with left IFJ activations.** (p < 0.001, FDR corrected), 1 Thalamus (predominantly medial dorsal nucleus), 2 Putamen.

There were bilateral co-activations in the PPC surrounding the intraparietal sulcus (IPS) mainly in the inferior and superior parietal lobule and partially extending into the precuneus (see Figure 1d). Clusters of significant activation likelihood were also observed surrounding the cerebellar tentorium (Figure 1d) extensively overlapping with the fusiform gyrus. Yet the peak coordinates were mainly located in the cerebellum according to the atlas labels. Subcortical areas associated with left IFJ activations are depicted in Figure 2. Right IFJ associations are comparable. The submaxima of the subcortical clusters were located in the putamen and the thalamus, mainly the medial dorsal nucleus. For a detailed description of all clusters and their submaxima significantly co-activated with the IFJ across studies see Tables 1 and 2.

**Table 1:** Brain areas (cluster-information and peak voxels) co-activated with the left IFJ

**Anatomical label**	**BA**	**(Sub-)Maxima coordinates**			**ALE**
		**x**	**y**	**z**	
Cluster 1 (180 contributing experiments, volume: 26888 mm^3^, weighted center: x = -44, y = 9, z = 27)					
Left Inferior Frontal Gyrus	9	−46	4	32	0.485
Left Insula		−32	20	6	0.159
Left Middle Frontal Gyrus	46	−46	24	22	0.114
Left Precentral Gyrus	4	−46	−10	48	0.096
Left Precentral Gyrus	44	−50	10	6	0.083
Left Precentral Gyrus	6	−26	−12	54	0.073
Left Inferior Frontal Gyrus	46	−44	32	10	0.054
Cluster 2 (108 contributing experiments, volume: 14600 mm^3^, weighted center: x = -33, y = -55, z = 42)					
Left Inferior Parietal Lobule	7	−30	−56	42	0.137
Left Superior Parietal Lobule	7	−24	−66	42	0.119
Left Inferior Parietal Lobule	40	−42	−42	38	0.092
Cluster 3 (114 contributing experiments, volume: 13808 mm^3^, weighted center: x = -1, y = 7, z = 49)					
Left Medial Frontal Gyrus	6	−2	2	54	0.192
Right Medial Frontal Gyrus	32	2	10	46	0.169
Cluster 4 (63 contributing experiments, volume: 6360 mm^3^, weighted center: x = 46, y = 8, z = 32)					
Right Inferior Frontal Gyrus	9	46	6	30	0.117
Right Inferior Frontal Gyrus	9	46	16	26	0.093
Cluster 5 (48 contributing experiments, volume: 5888 mm^3^, weighted center: x = -5, y = -8, z = 10)					
Left Putamen		−20	0	8	0.091
Left Thalamus		−12	−16	10	0.081
Right Putamen		16	0	10	0.069
Right Thalamus (Medial Dorsal Nucleus)		8	−18	10	0.056
Cluster 6 (38 contributing experiments, volume: 3808 mm^3^, weighted center: x = -41, y = -55, z = -16)					
Left Cerebellum (Posterior Lobe, Declive)		−38	−64	−14	0.070
Left Inferior Temporal Gyrus	20	−50	−52	−12	0.063
Left Cerebellum (Anterior Lobe, Culmen)		−40	−50	−20	0.062
Left Cerebellum (Anterior Lobe, Culmen)		−28	−56	−24	0.048
Cluster 7 (37 contributing experiments, volume: 3656 mm^3^, weighted center: x = 34, y = -52, z = 43)					
Right Inferior Parietal Lobule	40	36	−48	42	0.094
Right Precuneus	7	28	−64	38	0.055
Cluster 8 (32 contributing experiments, volume: 2488 mm^3^)					
Right Insula		32	18	10	0.095
Cluster 9 (16 contributing experiments, volume: 1232 mm^3^)					
Right Middle Frontal Gyrus	6	28	−8	54	0.067
Cluster 10 (11 contributing experiments, volume: 752 mm^3^)					
Left Inferior Occipital Gyrus	18	−28	−88	−8	0.067
Cluster 11 (9 contributing experiments, volume: 744 mm^3^)					
Right Middle Frontal Gyrus	46	40	32	24	0.065
Cluster 12 (10 contributing experiments, volume: 480 mm^3^)					
Left Middle Temporal Gyrus	22	−50	−40	10	0.058
Cluster 13 (6 contributing experiments, volume: 344 mm^3^)					
Left Middle Temporal Gyrus	22	−58	−34	4	0.060
Cluster 14 (4 contributing experiments, volume: 216 mm^3^)					
Right Cerebellum (Posterior Lobe, Declive)		22	−62	−22	0.054

p < 0.001, FDR corrected, cluster-size-threshold: 200 mm^3^.

**Table 2:** Brain areas (cluster-information and peak voxels) co-activated with the right IFJ

**Anatomical label**	**BA**	**(Sub-)Maxima coordinates**			**ALE**
		**x**	**y**	**z**	
Cluster 1 (105 contributing experiments, volume: 21336 mm^3^, weighted center: x = -42, y = 8, z = 25)					
Left Precentral Gyrus	6	−42	2	32	0.124
Left Inferior Frontal Gyrus	9	−48	6	30	0.120
Left Insula		−32	16	10	0.105
Left Middle Frontal Gyrus	6	−26	−10	54	0.075
Left Inferior Frontal Gyrus	47	−44	24	−2	0.059
Left Insula		−48	12	2	0.057
Left Precentral Gyrus	44	−50	10	8	0.057
Left Middle Frontal Gyrus	9	−42	30	26	0.051
Cluster 2 (131 contributing experiments, volume: 19224 mm^3^, weighted center: x = 44, y = 10, z = 26)					
Right Inferior Frontal Gyrus	9	46	6	32	0.397
Right Insula		32	18	8	0.108
Right Middle Frontal Gyrus	46	40	34	24	0.075
Right Insula		48	10	6	0.062
Right Insula		36	4	8	0.042
Cluster 3 (82 contributing experiments, volume: 11096 mm^3^)					
Left Superior Frontal Gyrus	6	−2	4	48	0.156
Cluster 4 (63 contributing experiments, volume: 8952 mm^3^, weighted center: x = 30, y = -58, z = 42)					
Right Superior Parietal Lobule	7	26	−66	42	0.091
Right Inferior Parietal Lobule	40	38	−48	42	0.087
Cluster 5 (53 contributing experiments, volume: 5896 mm^3^, weighted center: x = -32, y = -58, z = 43)					
Left Superior Parietal Lobule	7	−28	−62	44	0.094
Left Inferior Parietal Lobule	40	−40	−50	44	0.075
Cluster 6 (28 contributing experiments, volume: 2736 mm^3^, weighted center: x = -14, y = -10, z = 9)					
Left Thalamus (Medial Dorsal Nucleus)		−10	−18	10	0.074
Left Putamen		−20	0	8	0.059
Cluster 7 (20 contributing experiments, volume: 2280 mm^3^, weighted center: x = 10, y = -12, z = 10)					
Right Thalamus (Medial Dorsal Nucleus)		8	−18	10	0.071
Right Putamen		18	2	10	0.052
Cluster 8 (22 contributing experiments, volume: 2056 mm^3^)					
Right Precentral Gyrus	6	32	−8	56	0.070
Cluster 9 (3 contributing experiments, volume: 408 mm^3^)					
Right Inferior Temporal Gyrus	19	44	−60	−6	0.056
Cluster 10 (6 contributing experiments, volume: 280 mm^3^)					
Right Superior Temporal Gyrus		58	−38	18	0.046

p < 0.001, FDR corrected, cluster-size-threshold: 200 mm^3^.

**Figure 3:**
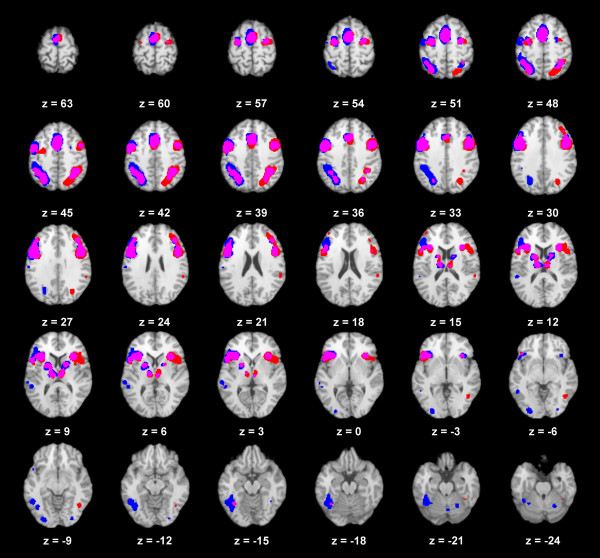
**Comparison of co-activation patterns with the left (blue) and right (red) IFJ.** (p < 0.001, FDR corrected) and overlapping areas (magenta).

The locations of the main frontal, parietal, insular and subcortical co-activations are largely comparable in both cerebral hemispheres for both IFJ seeds. The cluster in the left occipito-temporal area was marked in the left hemisphere in conjunction with the left IFJ. The extent of lateralization in the data is visualized in Figure 3.

### Intrinsic functional connectivity results

Resting-state analyses yielded a set of brain regions exhibiting correlated activity with the IFJ widely overlapping in all seeds and with the meta-analytic results: bilateral DLPFC, Broca’s area (left seeds only), medial frontal cortex, anterior insula, PPC, occipito-temporal junction, premotor cortex, striatum and thalamus (mainly medial dorsal nucleus). The VLPFC was included in the maps of suprathreshold voxels yet it could not be identified as an independent local maximum (Table 3). Additionally, consistent functional connectivity across seeds was observed with the anterior cingulate cortex and further parts of the cerebellum not covered in the meta-analysis (Additional file 1: Table S1).

**Table 3:** Peak coordinates of brain areas exhibiting correlated activity with the IFJ in the resting-state fMRI analysis that correspond to the cognitive control network observed in the MACM analysis

	**Seed 1**	**Seed 2**	**Seed 3**	**Seed 4**
	**(left IFJ)**	**(right IFJ)**	**(left IFJ)**	**(right IFJ)**
	**x, y, z**	**x, y, z**	**x, y, z**	**x, y, z**
Left IFJ	−46, 7, 33	−48, 3, 33	−42, 5, 33	−48, 1, 32
	(60, -5, 30)			
Right IFJ	45, 8, 33	49, 8, 36	41, 7, 33	41, 7, 33
	52, 12, 33			
Left DLPFC	−44, 35, 12	−45, 37, 16	−44, 35, 12	−42, 28, 26
		(-44, 19, 29)	(-42, 41, 6)	-45, 35, 18
Right DLPFC	47, 30, 26	45, 30, 22	43, 26, 24	45, 30, 22
	(45, 45, 4)	(44, 41, 9)		
Right VLPFC	(36, 38, -4)		(36, 38, -4)	
Broca’s area	−51, 14, 3		−49, 16, 1	
Medial Frontal Cortex	−5, 14, 51	4, 14, 51	−5, 14, 51	4, 12, 52
Left Anterior Insula	−27, 17, 5	−32, 17, 4	−29, 17, 4	−32, 17, 4
Right Anterior Insula	29, 21, 3	27, 21, 3	29, 21, 3	29, 21, 3
Left PPC	−44, -43, 41	−39, -44, 37	−42, -45, 41	−42, -41, 39
	-31, -56, 38		-31, -56, 38	
Right PPC	43, -42, 44	43, -44, 46	28, -62, 40	37, -43, 42
	28, -62, 40	35, -51, 43	41, -41, 42	45, -40, 45
		30, -72, 30		33, -51, 41
				(30, -74, 28)
				(13, -68, 50)
Left Occipito-Temporal Junction	−51, -51, -14	−51, -57, -11	−51, -51, -14	−49, -55, -11
Right Occipito-Temporal Junction	55, -48, -8	53, -50, -5	53, -46, -10	51, -50, -3
Left Premotor Cortex	−30, -3, 59	−26, -3, 58	−31, -3, 59	−26, -4, 48
		-37, -9, 55		-31, -11, 42
		-31, -11, 42		-26, -3, 56
		-26, -4, 50		
		11, -4, 65		
Right Premotor Cortex	30, -2, 59	33, -5, 58	30, -2, 59	35, -7, 56
	34, -5, 53		34, -5, 53	28, -5, 60
Left Thalamus	−9, -15, 10		−9, -15, 10	
			-5, -14, 17	
Right Thalamus		10, -14, 9		10, -14, 9
Left Striatum	−18, 4, 6	−21, 0, 8	−14, 5, 15	−18, -5, 16
	-14, 5, 15	-16, -3, 16	10, 5, 14	-14, 1, 13
	-23, -3, 0			-21, 0, 8
Right Striatum	10, 7, 12	19, 0, 6		19, 0, 6
		4, 1, 13		14, -1, 17
		14, -1, 17		4, 1, 13

p < 0.001, FWE corrected, cluster-size-threshold: 10 voxels), coordinates in parentheses only coarsely match the coordinates observed in the meta-analysis.

## Discussion

### Network subregions and correspondence with prior network definitions

The lateral frontal clusters of co-activation with the IFJ are formed by a set of confluent separate frontal cortical areas that have been well-characterized in the literature: A group of co-activation peaks rostral to the IFJ, predominantly assigned to the middle fontral gyrus in this analysis highly corresponds to an earlier definition of the mid-dorsolateral prefrontal cortex (mid-DLPFC) as discussed as constituent of a network subserving multiple cognitive demands [37] and other common definitions of dorsolateral prefrontal cortex (DLPFC) in stereotaxic space, e.g. [38,39]. Another consistent finding was a common co-activation of the IFJ with a set of peak activations in the mid-ventrolateral prefrontal cortex (mid-VLPFC) extending to the anterior insula [37], partially overlapping with other prior definitions of mid-VLPFC and anterior VLPFC [40]. Co-activations in BA 44 resemble the location of Broca’s area in terms of a coordinate-based definition [41].

Observed peak coordinates comprise a range of precentral co-activations. One of these peaks is located close to the frontal eye field. However it better corresponds to an adjacent region that has previously been associated with visuomotor hand conditional activity [42]. Thus in summary, the precentral peaks observed are presumably to some extent more directly associated with task execution than the other observed co-activations, as a range of studies included in this meta-analysis adopted task vs. baseline contrasts. The oberved association in our rs-fc analysis (Table 3) point even to a direct association in terms of neuronal activity.

In addition to these lateral frontal areas the network observed in this meta-analysis comprises medial frontal, posterior parietal and inferior posterior cortical areas (Tables 1 and 2, Figure 1). In this respect results are in line with findings based on other approaches to the study of functional brain networks that did however not directly focus on the IFJ: Similar activations of the medial wall have been observed by Duncan et. al. in their analyses focusing on frontal networks subserving multiple cognitive demands [37]. Similarly, mainly fronto-parietal networks are common findings in functional connectivity analyses based on resting-state fMRI acquisition: A set of lateral frontal (including precentral), insular, medial frontal, posterior parietal and inferior posterior foci form the ‘task-positive network’ in a study by Fox et al. [43]. A similar network has been observed with an exploratory approach based on independent component analyses as well in resting-state fMRI data as in data derived from the BrainMap database analyzed without constraints regarding functional areas or specific tasks [10].

Our results are highly concordant with the involvement of the IFJ in the cognitive control network (CCN) proposed by Cole et. al. [9]. All its components were found to be significantly co-activated with the IFJ: the DLPFC, pre-SMA, anterior insular cortex and PPC as well as matching aspects of the dorsal premotor cortex as far as coordinates are concerned. In contrast, in that study activations in Broca’s area were not tightly coupled with the CCN. Thus, co-activations of the IFJ with Broca’s area in our analysis could be interpreted as an evidence of a relation to additional language processing demands in the tasks included without representing a direct functional connection. Yet rs-fc results support a more direct links in terms of coherent neuronal activity (Table 3).

A recent task-based meta-analysis of cognitive control identified a comparable fronto-parietal CCN including the IFJ. Yet it was labeled as part of the inferior frontal gyrus (IFG) based on its Talairach coordinates (-42, 4 ,30 and 44, 6 32) in that case. The thalamus was also identified in an overall analysis across task-domains, yet it did not survive a formal conjunction analysis of different sub-domains of the construct of cognitive control [11].

Moreover, as a rather consistent finding we observed parallel activations of the IFJ with the basal ganglia (mainly the lentiform nucleus) and the thalamus. Regarding thalamic activation there might be a pitfall in ALE analyses related to the more spherical structure of its nuclei compared to rather flat cortical areas: Thalamic activations arising from different nuclei may be concatenated to a single cluster or even lead to a common peak location near midline. This is especially problematic as the activations observed here are finally assigned to the medial-dorsal nucleus (MDN), indeed a near-midline structure. However, thalamic co-activations form rather separate sub-clusters in both thalamic hemispheres (see Figure 2). In addition it is the MDN that has in previous studies been closely associated with the prefrontal cortex and higher cognitive functions in contrast to more lateral thalamic nuclei: Connections of the MDN with prefrontal brain areas have been observed using diffusion-MRI based tractography in humans including the DLPFC [44,45] and primary fMRI functional connectivity analyses [46]. This notion is also supported by animal studies [47]. This applies to the thalamic peaks observed here as well: After conversion of the coordinates into MNI space using the corresponding tool provided in GingerALE [28,48], the left thalamic peak exhibited a probability of 0.87 and the right thalamic peak of 0.79 to be connected with the pre-frontal cortex (without differentiation of subdivisions) according to a probabilistic human tractography atlas based on diffusion-MRI [49-51]. The probability of direct structural connectivity to the posterior parietal cortex was however nearly non-existent, reflecting the fact that diffusion tractography only detects direct fiber connections and not polysynaptical connectivity in functional networks. Human lesion data suggests, that executive dysfunction may arise from combined lesioning of several thalamic structures including the MDN [52].

### Comparison of MACM and resting-state results

Meta-analytic results were mostly confirmed using an analysis of intrinsic BOLD signal fluctuations in a presumably independent, publicly available dataset. However, there were some distinct differences: The VLPFC was less clearly identifiable in the resting-state analysis. It was present in the correlation maps but it was not marked as a distinct local maximum. The definition of local maxima in the rs-fc analysis was however constrained by a distance criterion (8 mm). As the VLPFC is wedged in Broca’s area in the left hemisphere and the anterior insula as well as the DLPFC in both hemispheres it might have been missed for that reason.

The ACC has been extensively studied as a region crucial for cognitive control processes [53]. Therefore its identification in the resting-state analysis is in line with previous findings. More inferior parts of the cerebellum identified in the rs-fc analysis might have been missed in the MACM analysis because this inferior region is not usually covered in many functional neuroimaging studies. Superior and middle temporal locations were only identified quite inconsistently when comparing different analysis strategies and can therefore not be considered a verified finding in this study.

Finally there were some pre- and postcentral areas of significant functional connectivity in the analysis of resting-state data that were not observed in the MACM analysis.

In contrast to prior findings in resting-state fMRI analyses based on spatial independent component analyses (ICA) [10] the network observed here appears more interhemispherically connected and additionally overlaps with a fronto-insular component. This may be related to a possible advantage of the meta-analytic connectivity modeling approach adopted here: classical definitions of functional connectivity are based on the analysis of a tight temporal coupling of neurophysiological events [54]. In contrast, functional connectivity in terms of MACM can be interpreted as remote brain areas cooperating in dealing with a task without necessarily exhibiting highly temporally correlated activity. Thus if two rather independent networks in terms of direct structural connectivity or classical functional connectivity are parallel recruited due to comparable task demands, these networks can potentially be identified as one coherent network by MACM [13]. This clear differentiation is however limited by our seed-based rs-fc analysis: Though exhibiting a certain degree of asymmetry, resting-state networks were not limited to the seed’s hemisphere. The main difference might thus arise from different analysis strategies of rs-fc analyses (with spatial ICA emphasizing spatial independence of networks).

There are different possible explanations for the fact that more regions were connected to the IFJ in the analysis of the resting-state dataset compared to the MACM results: Statistical power of both approaches is most likely different. In addition to some baseline-comparisons the BrainMap database contains coordinates from many well-controlled fMRI contrasts to delineate specific behavioral processes by including associated functions (e.g. stimulus-perception and motor responses) in control conditions. The additional correlations in the resting-state data may therefore also represent meaningful and necessary connections of the actual CCN components to brain areas relevant for direct interaction with the environment.

Though the results of both methods are comparable the occasional differences of resting-state and MACM results point to the critical fact that current converging methods applied in the study of complex brain networks may oversimplify the actual functional organization of the human brain as they may not optimally account for the internal organization of such networks and their complex interdependences. It is a notable finding in this context that analyzing fMRI data with an increased temporal resolution using temporal ICA Smith et al. recently reported temporally-independent functional modes of spontaneous brain activity that overlapped with each other and networks known from conventional (seed-correlation or spatial ICA) analyses [55].

### Functional implications

As stated in the introduction the IFJ has been studied as a specific brain area in task-based fMRI and meta-analyses limited to a few task domains. Results can be summarized as an involvement of the IFJ in three main component processes of cognitive control (task switching, inhibitory control and working memory) [1-8].

The functional significance of similar fronto-parietal networks as observed in this study has explicitly or implicitly been assessed in numerous often highly specific task-based studies. As reported above, a recent meta-analysis has accumulated such findings based on the BrainMap database [11]: In that meta-analysis cognitive control was operationalized as initiation, inhibition, working memory, flexibility, planning and vigilance. Therefore for each of these sub-domains a set of established tasks (like Flanker, Go/No-Go, Antisaccade, Simon and Stroop tasks for inhibition) was defined and the studies included in the final analyses were restricted to those using these a priori defined tasks. A core network was observed in a conjunction analysis of flexibility, inhibition and working memory that highly overlapped with the CCN observed in the MACM analysis reported here. Thus results point to an involvement of the CCN in all of these functions in a rather unspecific way and to a connection of the IFJ with these other regions in this context.

In contrast to these previous meta-analytic approaches providing information about the IFJ [3] or the CCN [11] the analysis reported in this article is conceptually different (1) in that it is not a priori limited to the context of cognitive control and (2) in that it starts from the IFJ as a previously defined specific location in the brain and therefore adds specificity to the knowledge about this set of connections. Although our analysis aimed at studying connectivity, this framework can also be used to explore functional meanings of the IFJ and the CCN using an analogous approach: The BrainMap database contains structured information (hierarchical meta-data) about behavioral aspects represented in the reported contrasts. Lancaster et al. have reported an automated behavioral analysis based on these meta-data that allows ROI-based searches [56]. Queries regarding the IFJ and the whole network observed here (Additional file 1: Table S2) give a rough estimation of functions associated with the specific coordinate definitions and network maps in this study. They show a statistically significant association with cognitive processes including (working) memory, inhibition and attention but among others also language processing (left hemisphere) and perceptive processes presumably involved in some of the chosen fMRI paradigms.

There is evidence in the meta-analytic results on CCN functions by Niendam et. al. [11] that in addition to the rather unspecific involvement of the CCN core regions additional areas are recruited in a sub-domain-specific manner. This finding is compatible with the recent meta-analytic and task-based finding that within the frontal cortex the IFJ is generally involved in the cognitive control subdomain of switching / flexibility but together with other lateral and medial frontal regions which are recruited more specifically [7,8]. This in turn is in some way reminiscent of the assumption of a hierarchical organization of the rostro-caudal axis of the frontal lobes [57].

Taken together but potentially limited by the power to detect the involvement of certain brain regions in the different approaches reported the findings seem to support the notion that the IFJ is rather specifically involved in brain systems playing an important role in cognitive control compared to other aspects of brain function.

### Limitations

A potential limitation of the MACM approach may arise from the fact that, unlike for example in resting-state fMRI approaches to functional connectivity, results, though including several different functional domains, may be influenced by the overall distribution of tasks in the BrainMap database and correspondingly the distribution of tasks adopted by the whole functional neuroimaging community. However, as discussed above, our results are in line with recent literature regarding IFJ connectivity and a different approach to MACM [58] adopted in the NeuroSynth project (http://www.neurosynth.org/) yields qualitatively comparable results regarding IFJ connectivity, thus at least we suppose that there is no specific bias regarding the BrainMap database and studies included.

Recently it has been argued that the IFJ is functionally dissociated from the directly adjacent posterior part of the inferior frontal gyrus (pIFG) [59]. We have not directly observed this dissociation in terms of different peaks in our analysis. The used cuboid-shaped ROIs were based on prior literature regarding IFJ location in stereotaxic space. However, irregularly shaped ROIs might better conform to the IFJ as a functional brain area and help clarify this issue.

The selection of coordinates most exactly representing the IFJ in stereotaxic space is still a matter of debate. For the meta-analysis we aimed at high specificity regarding the IFJ as a distinct functional brain region without accidentally including other functionally defined areas in our seed. We therefore selected a relatively small ROI based on a large body of literature aggregated in a review based on a number of single studies and meta-analytical data [1]. Yet there is recent data that suggests that the IFJ may be located more medial in some subjects [2]. The meta-analysis might thus have missed some studies reporting IFJ peaks just outside the ROI borders. We addressed this issue by using two different ROI definition strategies in the confirmatory analysis based on resting-state functional connectivity. Results were not qualitatively different regarding the identification of the main constituent brain areas of the CCN) when comparing the more lateral and more medial ROI in each hemisphere.

Though the main finding of this study is a considerably specific connection of the IFJ with brain regions previously characterized as a network engaged in cognitive control from a systems neuroscience perspective, it cannot be reliably deduced from these results that the CCN is indeed ‘only’ involved in cognitive control from a classical neuropsychological perspective. This would be some kind of problematic reverse inference [60]. Though the additional analysis of BrainMap meta-data reported shows a significant association of the IFJ as well as the whole network with ‘cognitive’ tasks, a disjunctive analysis of ‘cognitive control’ in a strict sense does not seem to be feasible in this framework, especially due to the fact that the above-mentioned definition of cognitive control partially overlaps with different categories in the BrainMap meta-data. The behavioral analysis in this framework is also limited by the fact that the set of studies which the analysis is based on overlaps with previous function-guided meta-analyses and also includes, as a minority, studies that have originally led to the fundamental assumption that this specific brain location in the inferior frontal junction area is involved in cognitive control.

## Conclusions

Using a metaanalytic connectivity modeling (MACM) approach with data from the BrainMap database of functional neuroimaging studies we characterized the functional connectivity profile of the inferior frontal junction area (IFJ) in terms of co-activations. Based on a large dataset of published peak activations our results confirm the notion that the IFJ is involved in a cognitive control network (CCN). The CCN comprises the dorsolateral prefrontal cortex, anterior insula (and neighboring ventrolateral prefrontal cortex), the medial frontal cortex/pre-SMA, parts of the dorsal premotor cortex and the posterior parietal cortex. In addition we identified connectivity of the IFJ with subcortical areas mainly pertaining to the medial dorsal nucleus of the thalamus and an inferior posterior region surrounding the cerebellar tentorium that cannot unequivocally be ascribed to the cerebellum or occipito-temporal junction based on the Talairach atlas. Assigning these locations to the CCN is compatible with prior observations mainly in resting-state fMRI and structural connectivity analyses. Other co-activations most consistently observed include language and motor-related locations. Results were largely confirmed in an additional resting-state fMRI analysis. However, using this approach, the VLPFC was less clearly identified and the CCN was complemented by the anterior cingulate cortex in line with previous observations.

The CCN involving the IFJ has been proposed previously by using task-based and resting-state fMRI data in a number of subjects [9]. Most of the larger studies on fronto-parietal networks relevant for cognition do not denominate the IFJ explicitly. Thus, results reported here establish the formal link between previous work on IFJ functionality and studies focusing on the overall organization of cognitive brain networks on a high formal level of (meta-analytical) evidence. It has to be emphasized however that models like the CCN are only approximations of the human brain’s functional organization and cannot fully capture interdependencies between and specialization within networks.

Still, in light of results suggesting a role of impaired IFJ functionality in early dementia together with this information of functional connectivity of the IFJ in the CCN, the IFJ seems to be a promising starting point to investigate the cognitive control network in further studies and in particular in clinical populations.

## Abbreviations

ACC: Anterior cingulate cortex; ALE: Activation likelihood estimation; BA: Brodmann area; CCN: Cognitive control network; DLPFC: Dorsolateral prefrontal cortex; FCP: 1000 Functional Connectomes Project; FDG-PET: Fluorodeoxyglucose positron emission tomography; FDR: False-discovery rate; fMRI: Functional magnetic resonance imaging; FWE: Family-wise error-rate; ICA: Independent component analysis; IFJ: Inferior frontal junction; IPS: Intraparietal sulcus; MACM: Metaanalytic connectivity modelling; MDN: Medial-dorsal nucleus of the thalamus; MNI: Montreal Neurological Institute; pIFG: Posterior part of the inferior frontal gyrus; PPC: Posterior parietal cortex; pSMA: Pre-supplementary motor area; ROI: Region of interest; rsFC: Resting-state functional connectivity; VLPFC: Ventrolateral prefrontal cortex.

## Competing interests

The authors declare that there are no potential competing interests.

## Authors’ contributions

BS conceived the study and analyzed the data. BS and BP participated in study design, interpretation of the results and drafted the manuscript. Both authors approved the final manuscript.

## Supplementary Material

Additional file 1: Table S1 Peak coordinates and Talairach atlas labels of brain areas exhibiting correlated activity with the IFJ in the resting-state fMRI analysis that do not conclusively correspond to the cognitive control network observed in the MACM analysis (p < 0.001, FWE corrected, cluster-size-threshold: 10 voxels)., **Table S2 –** behavioral analysis of BrainMap-data on IFJ and CCN activations, **List of References** – 139 articles initially identified for the left IFJ, 111 articles initially identified for the right IFJ. Click here for file
