# Supplementary material for: Functional connectivity profile of the human inferior frontal junction: involvement in a cognitive control network
Source: BMC Neurosci. 2012 Oct 3;13:119. doi: 10.1186/1471-2202-13-119 (PMC3582543; doi:10.1186/1471-2202-13-119)
Supplement: Additional file 1: Table S1 — Peak coordinates and Talairach atlas labels of brain areas exhibiting correlated activity with the IFJ in the resting-state fMRI analysis that do not conclusively correspond to the cognitive control network observed in the MACM analysis (p < 0.001, FWE corrected, cluster-size-threshold: 10 voxels)., Table S2 – behavioral analysis of BrainMap-data on IFJ and CCN activations, List of References – 139 articles initially identified for the left IFJ, 111 articles initially identified for the right IFJ. [file 1471-2202-13-119-S1.pdf]

Sundermann B, Pfleiderer B

Functional connectivity profile of the human inferior frontal junction: involvement in a cognitive control network

Supplementary Table 1

peak coordinates and Talairach atlas labels of brain areas exhibiting correlated activity with the IFJ in the resting-state fMRI analysis that do not conclusively correspond to the cognitive control network observed in the MACM analysis ($p < 0.001$, FWE corrected, cluster-size-threshold: 10 voxels)

Anatomical label	BA	x	y	z
Correlation with seed 1 (left IFJ)				
Left Cingulate Gyrus	24	-5	0	29
Right Cingulate Gyrus	24	4	-3	31
Right Superior Temporal Gyrus	22	63	-37	18
Left Claustrum		-34	-4	7
Left Putamen		-31	-14	-7
Left Anterior Cingulate	32	-17	39	-7
Left Claustrum		-29	-25	9
Right Postcentral Gyrus	3	60	-20	36
(Right Hemisphere)	10	44	44	-3
Right Postcentral Gyrus	1	63	-15	26
Right Postcentral Gyrus	2	39	-24	33
Right Postcentral Gyrus	2	60	-30	40
Right Middle Frontal Gyrus	11	21	37	-6
Right Cerebellum (Posterior Lobe)		8	-74	-28
Right Cerebellum (Posterior Lobe)		25	-65	-46
Right Cerebellum (Posterior Lobe)		23	-61	-30
Left Cerebellum (Posterior Lobe)		-10	-72	-30
Right Superior Temporal Gyrus	42	65	-25	10

Left Inferior Frontal Gyrus	47	-21	17	-18
(Right Hemisphere)		-25	-64	-49
Correlation with seed 2 (right IFJ)				
Left Cingulate Gyrus	24	-5	1	31
Left Cingulate Gyrus	24	2	1	30
Right Superior Temporal Gyrus		47	-43	19
Right Inferior Frontal Gyrus	47	20	35	-7
Right Claustrum		36	-2	8
Right Putamen		29	-14	-4
Right Red Nucleus		6	-18	-5
Right Inferior Frontal Gyrus	47	16	15	-19
Right Claustrum		27	-23	13
Right Superior Temporal Gyrus	22	38	-55	12
Left Red Nucleus		-5	-18	-5
Left Middle Occipital Gyrus	19	-35	-82	19
Left Cerebellum (Posterior Lobe)		-10	-70	-28
Left Cerebellum (Posterior Lobe)		-23	-64	-47
Left Cerebellum (Posterior Lobe)		-23	-61	-29
Left Right Cingulate Gyrus	24	12	-12	34
Left Claustrum		-38	-4	7
Right Cerebellum (Posterior Lobe)		20	-65	-47
Left Cingulate Gyrus	24	-14	-20	35
Right Hypothalamus		1	-2	-17
Correlation with seed 3 (left IFJ)				
Left Cingulate Gyrus	24	-5	0	29

Right Cingulate Gyrus	24	4	-3	31
Right Superior Temporal Gyrus	42	64	-29	10
Left Putamen		-31	-14	-7
Left Anterior Cingulate	32	-17	39	-7
Left Inferior Frontal Gyrus	47	-21	17	-18
Right Postcentral Gyrus	2	39	-24	33
Right Middle Frontal Gyrus	10	42	44	-5
Right Postcentral Gyrus	3	50	-16	34
Right Postcentral Gyrus	3	60	-18	32
Right Postcentral Gyrus	1	63	-15	26
Right Precentral Gyrus	6	58	-7	32
Right Postcentral Gyrus	2	60	-30	40
Right Inferior Frontal Gyrus	47	23	35	-6
Left Superior Temporal Gyrus	39	-42	-49	8
Left Cingulate Gyrus	24	-13	-12	34
Right Cerebellum (Posterior Lobe)		10	-72	-28
Right Cerebellum (Posterior Lobe)		25	-66	-47
Right Cerebellum (Posterior Lobe)		25	-59	-30
Left Claustrum		-34	-4	7
Left Claustrum		-29	-25	10
Left Cerebellum (Posterior Lobe)		-10	-72	-28
Correlation with seed 4 (right IFJ)				
Left Cingulate Gyrus	24	-7	1	31
Right Cingulate Gyrus	24	2	1	30
Right Superior Temporal Gyrus		47	-43	19
Right Claustrum		34	0	5

Right Putamen		31	-14	-6
Right Inferior Frontal Gyrus	47	20	33	-7
Left Cingulate Gyrus	24	-14	-20	35
Right Cingulate Gyrus	31	13	-33	36
Right Cingulate Gyrus	24	12	-18	33
Right Red Nucleus		6	-20	-3
Right Claustrum		27	-22	13
Right Cingulate Gyrus	31	8	-45	43
Right Inferior Frontal Gyrus	47	16	14	-19
Right Middle Temporal Gyrus	37	39	-63	12
Left Claustrum		-36	-4	7
Left Superior Occipital Gyrus	19	-29	-79	25
Left Middle Occipital Gyrus	19	-35	-82	19
Left Cerebellum (Posterior Lobe)		-23	-64	-47
Left Cerebellum (Posterior Lobe)		-10	-70	-28
Left Cerebellum (Anterior Lobe)		-25	-59	-29
Left Putamen		-32	-14	-7
Right Cerebellum (Posterior Lobe)		20	-65	-47
Right Hypothalamus		1	-2	-17
Left Subthalamic Nucleus		-10	-14	-3
Left Red Nucleus		-5	-20	-3

Supplementary Table 2

Behavioral analysis of BrainMap-data on IFJ and CCN activations using an automated method based on BrainMap metadata, introduced by Lancaster et al. (Lancaster JL, Laird AR, Eickhoff SB, Martinez MJ, Fox PM, Fox PT: Automated regional behavioral analysis for human brain images. Front Neuroinform 2012, 6:23.) and implemented in Mango [http://ric.uthscsa.edu/mango/].

We searched these preformed results with 12 mm and 25 mm wide cubic ROIs centered at the IFJ in Talairach space (± 47, 5, 34) as well as masks of the whole connectivity map with the left or right IFJ as observed in this MACM analysis. Results were thresholded at Z = 3 as suggested by the authors of this method.

Category	Domain	Z-Score
Left IFJ, 12 mm		
Memory (Working)	Cognition	6.293
Language (Semantics)	Cognition	6.019
Attention	Cognition	5.714
Language (Speech)	Cognition	5.238
Memory (Explicit)	Cognition	4.625
Language (Orthography)	Cognition	4.372
Vision (Shape)	Perception	3.479
Language (Phonology)	Cognition	3.476
Other	Cognition	3.279
Other	Emotion	3.019
Left IFJ, 25 mm		
Language (Semantics)	Cognition	12.845
Memory (Working)	Cognition	12.036
Language (Speech)	Cognition	11.798
Language (Phonology)	Cognition	8.98
Attention	Cognition	8.389
Execution (Speech)	Action	7.037
Memory (Explicit)	Cognition	6.861
Language (Orthography)	Cognition	6.465
Language (Syntax)	Cognition	4.78
Execution (Other)	Action	4.643
Language (Other)	Cognition	4.309
Imagination	Action	4.138
Other	Cognition	3.948
Other	Emotion	3.857
Audition	Perception	3.5
Vision (Shape)	Perception	3.497
Space	Cognition	3.226

Reasoning	Cognition	3.137
Inhibition	Action	3.001

Right IFJ, 12 mm

Attention	Cognition	6.027
Memory (Working)	Cognition	5.654
Execution (Other)	Action	3.581
Language (Speech)	Cognition	3.109
Vision (Shape)	Perception	3.051
Memory (Explicit)	Cognition	3.03
Other	Emotion	3.019

Right IFJ, 25 mm

Attention	Cognition	8.513
Memory (Working)	Cognition	6.947
Execution (Other)	Action	6.488
Language (Speech)	Cognition	4.928
Execution (Speech)	Action	4.253
Memory (Explicit)	Cognition	4.162
Vision (Other)	Perception	3.945
Observation	Action	3.903
Inhibition	Action	3.745
Audition	Perception	3.5
Vision (Shape)	Perception	3.232
Space	Cognition	3.123
Anger	Emotion	3.078
Somesthesis (Other)	Perception	3.067

Whole network based on left IFJ seed

Memory (Working)	Cognition	36.855
Language (Semantics)	Cognition	34.253
Attention	Cognition	33.463
Language (Speech)	Cognition	30.915
Execution (Other)	Action	26.101
Memory (Explicit)	Cognition	24.188
Other	Cognition	22.153
Other	Emotion	20.529
Language (Phonology)	Cognition	19.682
Vision (Motion)	Perception	17.778
Language (Orthography)	Cognition	17.621

Inhibition	Action	17.323
Execution (Speech)	Action	16.502
Space	Cognition	16.443
Vision (Shape)	Perception	16.435
Somesthesis (Pain)	Perception	15.039
Reasoning	Cognition	12.93
Imagination	Action	12.475
Audition	Perception	12.196
Language (Other)	Cognition	11.186
Somesthesis (Other)	Perception	11.016
Vision (Other)	Perception	10.774
Language (Syntax)	Cognition	10.738
Observation	Action	9.809
Music	Cognition	9.349
Time	Cognition	7.822
Motor (Learning)	Action	7.176
Gustation	Perception	6.257
Social	Cognition	6.087
Preparation	Action	5.666
Soma	Cognition	5.375
Fear	Emotion	5.167
Anger	Emotion	5.092
Rest	Action	4.329
Vision (Color)	Perception	4.026
Disgust	Emotion	3.573
Sexuality	Interoception	3.426
Sleep	Interoception	3.224
Other	Interoception	3.118
Anxiety	Emotion	3.045
Sadness	Emotion	3.042

Whole network based on right IFJ seed

Memory (Working)	Cognition	36.904
Attention	Cognition	34.537
Language (Semantics)	Cognition	28.629
Execution (Other)	Action	26.795
Language (Speech)	Cognition	26.774
Memory (Explicit)	Cognition	22.148
Other	Cognition	21.577
Other	Emotion	20.614
Inhibition	Action	18.684
Vision (Motion)	Perception	18.403
Somesthesis (Pain)	Perception	18.069

Language (Phonology)	Cognition	17.505
Space	Cognition	17.205
Vision (Shape)	Perception	16.229
Execution (Speech)	Action	15.761
Language (Orthography)	Cognition	15.347
Audition	Perception	12.055
Imagination	Action	11.835
Vision (Other)	Perception	11.524
Reasoning	Cognition	11.483
Somesthesis (Other)	Perception	11.399
Music	Cognition	10.669
Observation	Action	10
Language (Syntax)	Cognition	9.405
Language (Other)	Cognition	8.809
Time	Cognition	8.339
Motor (Learning)	Action	7.243
Gustation	Perception	6.493
Social	Cognition	6.031
Preparation	Action	5.913
Fear	Emotion	5.371
Soma	Cognition	4.968
Anger	Emotion	4.791
Rest	Action	4.361
Vision (Color)	Perception	4.341
Bladder	Interoception	3.861
Sleep	Interoception	3.812
Sexuality	Interoception	3.691
Disgust	Emotion	3.68
Anxiety	Emotion	3.482
Sadness	Emotion	3.463
Other	Interoception	3.034

Supplementary List of References

A – 139 articles initially identified for the left IFJ

1. Akine Y, Kato M, Muramatsu T, Umeda S, Mimura M, Asai Y, Tanada S, Obata T, Ikehira H, Kashima H,Suhara T.: **Altered brain activation by a false recognition task in young abstinent patients with alcohol dependence.** Alcoholism: Clinical and Experimental Research 2007, **31**:1589-1597.

2. Balsamo L M, Xu B, Grandin C B, Petrella J R, Braniecki S H, Elliott T K,Gaillard W D.: **A functional magnetic resonance imaging study of left hemisphere language dominance in children.** Arch Neurol 2002, **59**:1168-1174.

3. Banich M T, Milham M P, Atchley R A, Cohen N J, Webb A G, Wszalek T M, Kramer A F, Liang Z P, Barad V, Gullett D, Shah C,Brown C.: **Prefrontal regions play a dominant role in imposing an attentional 'set': Evidence from fMRI.** Cognitive Brain Research 2000, **10**:1-9.

4. Banich M T, Milham M P, Jacobson B L, Webb A G, Wszalek T M, Cohen N J,Kramer A F.: **Attentional selection and the processing of task-irrelevant information: Insights from fMRI examinations of the Stroop task.** Prog Brain Res 2001, **134**:459-470.

5. Basho S, Palmer E D, Rubio M A, Wulfeck B,Muller R A.: **Effects of generation mode in fMRI adaptations of semantic fluency: Paced production and overt speech.** Neuropsychologia 2007, **45**:1697-1706.

6. Bedwell J S, Horner M D, Yamanaka K, Li X, Myrick H, Nahas Z,George M S.: **Functional neuroanatomy of subcomponent cognitive processes involved in verbal working memory.** Int J Neurosci 2005, **115**:1017-1032.

7. Binkofski F, Buccino G, Posse S, Seitz R J, Rizzolatti G,Freund H J.: **A fronto-parietal circuit for object manipulation in man: evidence from an fMRI-study.** Eur J Neurosci 1999, **11**:3276-3286.

8. Bohland J W,Guenther F H.: **An fMRI investigation of syllable sequence production.** Neuroimage 2006, **32**:821-841.

9. Brass M,Yves von Cramon D.: **The role of the frontal cortex in task preparation.** Cerebral Cortex 2002, **12**:908-914.

10. Brown S MMJ: **Activation of premotor vocal areas during musical discrimination.** Brain Cogn 2007, **63**:59-69.

11. Cabeza R, Dolcos F, Prince S E, Rice H J, Weissman D H,Nyberg L.: **Attention-related activity during episodic memory retrieval: A cross-function fMRI study.** Neuropsychologia 2003, **41**:390-399.

12. Camchong J, Dyckman K A, Chapman C E, Yanasak N E,McDowell J E.: **Basal ganglia-thalamocortical circuitry disruptions in schizophrenia during delayed response tasks.** Biol Psychiatry 2006, **60**:235-241.

13. Canli T, Omura K, Haas B W, Fallgatter A, Constable R T,Lesch K P.: **Beyond affect: A role for genetic variation of the serotonin transporter in neural actiation during a cognitive attention task.** Proceedings of the National Academy of Sciences 2005, **102**:12224-12229.

14. Cansino S, Maquet P, Dolan R J,Rugg M D.: **Brain activity underlying encoding and retrieval of source memory.** Cerebral Cortex 2002, **12**:1048-1056.

15. Carreiras M, Mechelli A, Estevez A,Price C J.: **Brain activation for lexical decision and reading aloud: Two sides of the same coin.** J Cogn Neurosci 2007, **19**:433-444.

16. Cato M A, Crosson B, Gokcay D, Soltysik D, Wierenga C E, Gopinath K, Himes N, Belanger H, Bauer R M, Fischler I S, Gonzalez-Rothi L,Briggs R W.: **Processing words with emotional connotation: An FMRI study of time course and laterality in rostral frontal and retrosplenial cortices.** J Cogn Neurosci 2004, **16**:167-177.

17. Chikazoe J, Jimura K, Asari T, Yamashita K, Morimoto H, Hirose S, Miyashita Y,Konishi S.: **Functional dissociation in right inferior frontal cortex during performance of go/no-go task.** Cerebral Cortex 2009, **19**:146-152.

18. Chikazoe J, Jimura K, Hirose S, Yamashita K, Miyashita Y,Konishi S.: **Preparation to inhibit a response complements response inhibition during performance of a stop-signal task.** Journal of Neuroscience 2009, **29**:15870-15877.

19. Coderre E L, Filippi C G, Newhouse P A,Dumas J A.: **The Stroop effect in kana and kanji scripts in native Japanese speakers: An fMRI study.** Brain Lang 2008, **107**:124-132.

20. Cunnington R, Windischberger C, Robinson S,Moser E.: **The selection of intended actions and the observation of others' actions: A time-resolved fMRI study.** Neuroimage 2006, **29**:1294-1302.

21. Curtis V A, Bullmore E T, Brammer M J, Wright I C, Williams S C R, Morris R G, Sharma T S, Murray R M,McGuire P K.: **Attenuated frontal activation during a verbal fluency task in patients with schizophrenia.** Am J Psychiatry 1998, **155**:1056-1063.

22. Curtis V A, Bullmore E T, Morris R G, Brammer M J, Williams S C R, Simmons A, Sharma T S, Murray R M,McGuire P K.: **Attenuated frontal activation in schizophrenia may be task dependent.** Schizophr Res 1999, **37**:35-44.

23. Damasio H, Grabowski T J, Tranel D, Boles Ponto L L, Hichwa R D,Damasio A R.: **Neural correlates of naming actions and of naming spatial relations.** Neuroimage 2001, **13**:1053-1064.

24. Dapretto M BSY: **Form and content: Dissociating syntax and semantics in sentence comprehension.** Neuron 1999, **24**:427-432.

25. David N, Bewernick B H, Cohen M X, Newen A, Lux S, Fink G R, Shah N J,Vogeley K.: **Neural representations of self versus other: Visual-spatial perspective taking and agency in a virtual ball-tossing game.** J Cogn Neurosci 2006, **18**:898-910.

26. Dehaene S, Naccache L, Cohen L G, Le Bihan D, Mangin J F, Poline J B,Riviere D.: **Cerebral mechanisms of word masking and unconscious repetition priming.** Nat Neurosci 2001, **4**:752-758.

27. Devlin J T, Matthews P M,Rushworth M F.: **Semantic processing in the left inferior prefrontal cortex: A combined functional magnetic resonance imaging and transcranial magnetic stimulation study.** J Cogn Neurosci 2003, **15**:71-84.

28. Dreher J C GJ: **Dissociating the roles of the rostral anterior cingulate and the lateral prefrontal cortices in performing two tasks simultaneously or successively.** Cerebral Cortex 2003, **13**:329-339.

29. Drobyshevsky A, Baumann S B,Schneider W.: **A rapid fMRI task battery for mapping of visual, motor, cognitive, and emotional function.** Neuroimage 2006, **31**:732-744.

30. Druzgal T J DM: **A neural network reflecting decisions about human faces.** Neuron 2001, **32**:947-955.

31. Ernst M, Nelson E E, Jazbec S, McClure E B, Monk C S, Leibenluft E, Blair J,Pine D S.: **Amygdala and nucleus accumbens in responses to receipt and omission of gains in adults and adolescents.** Neuroimage 2005, **25**:1279-1291.

32. Ferrandez A M, Hugueville L, Lehericy S, Poline J B, Marsault C,Pouthas V.: **Basal ganglia and supplementary motor area subtend duration perception: An fMRI study.** Neuroimage 2003, **19**:1532-1544.

33. Ferstl E C,Yves von Cramon D.: **Time, space and emotion: fMRI reveals content-specific activation during text comprehension.** Neurosci Lett 2007, **427**:159-164.

34. Fincham J M, Carter C S, van Veen V, Stenger V A,Anderson J R.: **Neural mechanisms of planning: a computational analysis using event-related fMRI.** Proceedings of the National Academy of Sciences 2002, **99**:3346-3351.

35. Fitzgerald P B, Srithiran A, Benitez J, Daskalakis Z Z, Oxley T J, Kulkarni J,Egan G F.: **An fMRI study of prefrontal brain activation during multiple tasks in patients with major depressive disorder.** Hum Brain Mapp 2008, **29**:490-501.

36. Frangou S, Kington J, Raymont V,Shergill S S.: **Examining ventral and dorsal prefrontal function in bipolar disorder: A functional magnetic resonance imaging study.** European Psychiatry 2008, **23**:300-308.

37. Fu S, Chen Y, Smith S M, Iversen S D,Matthews P M.: **Effects of word form on brain processing of written Chinese.** Neuroimage 2002, **17**:1538-1548.

38. G. KJ: **Anterior cingulate and prefrontal cortex activity in an FMRI study of trial-to-trial adjustments on the Simon task.** Neuroimage 2006, **33**:399-405.

39. Gaillard W D, Sachs B C, Whitnah J R, Ahmad Z, Balsamo L M, Petrella J R, Braniecki S H, McKinney C M, Hunter K, Xu B,Grandin C B.: **Developmental aspects of language processing: fMRI of verbal fluency in children and adults.** Hum Brain Mapp 2003, **18**:176-185.

40. Gould R L, Brown R G, Owen A M, Ffytche D H,Howard R J.: **fMRI BOLD response to increasing task difficulty during successful paired associates learning.** Neuroimage 2003, **20**:1006-1019.

41. Grosbras M H PT: **Brain networks involved in viewing angry hands or faces.** Cerebral Cortex 2006, **16**:1087-1096.

42. Gruber O,Yves von Cramon D.: **The functional neuroanatomy of human working memory revisited: Evidence from 3-T fMRI studies using classical domain-specific interference tasks.** Neuroimage 2003, **19**:797-809.

43. Gur R C, Turetsky B I, Loughead J, Waxman J, Snyder W, Ragland J D, Elliot M A, Bilker W B, Arnold S E,Gur R E.: **Hemodynamic response in neural circuitries for detection of visual target and novelty: An event-related fMRI study.** Hum Brain Mapp 2007, **28**:263-274.

44. Hanakawa T, Dimyan M A,Hallett M.: **Motor planning, imagery, and execution in the distributed motor network: A time-course study with functional MRI.** Cerebral Cortex 2008, **18**:2775-2788.

45. Hare T, Camerer C F,Rangel A.: **Self-control in decision-making involves modulation of the vmPFC valuation system.** Science 2009, **324**:646-648.

46. Harrington G S, Farias D, Davis C H,Buonocore M H.: **Comparison of the neural basis for imagined writing and drawing.** Hum Brain Mapp 2007, **28**:450-459.

47. Haslinger B, Erhard P, Altenmuller E, Hennenlotter A, Schwaiger M, von Einsiedel H G, Rummeny E, Conrad B,Ceballos-Baumann A O.: **Reduced recruitment of motor association areas during bimanual coordination in concert pianists.** Hum Brain Mapp 2004, **22**:206-215.

48. Hauk O, Davis M H, Kherif F,Pulvermuller F.: **Imagery or meaning? Evidence for a semantic origin of category-specific brain activity in metabolic imaging.** Eur J Neurosci 2009, **27**:1856-1866.

49. Hautzel H, Mottaghy F M, Schmidt D, Zemb M, Shah N J, Muller-Gartner H W,Krause B J.: **Topographic segregation and convergence of verbal, object, shape and spatial working memory in humans.** Neurosci Lett 2002, **26**:156-160.

50. Hazeltine E, Bunge S A, Scanlon M D,Gabrieli J D E.: **Material-dependent and material-independent selection processes in the frontal and parietal lobes: An event-related fMRI investigation of response competition.** Neuropsychologia 2003, **41**:1208-1217.

51. Honey G D, Bullmore E T, Soni W, Varatheesan M, Williams S C R,Sharma T S.: **Differences in frontal cortical activation by a working memory task after substitution of risperidone for typical antipsychotic drugs in patients with schizophrenia.** Proceedings of the National Academy of Sciences 1999, **96**:13432-13437.

52. Honey G D, Sharma T S, Suckling J, Giampietro V, Williams S C R,Bullmore E T.: **The functional neuroanatomy of schizophrenic subsyndromes.** Psychol Med 2003, **33**:1007-1018.

53. Ilg R, Vogeley K, Goschke T, Bolte A, Shah J N, Poppel E,Fink G R.: **Neural processes underlying intuitive coherence judgments as revealed by fMRI on a semantic judgment task.** Neuroimage 2007, **38**:228-238.

54. Ino T, Doi T, Kimura T, Ito J,Fukuyama H.: **Neural substrates of the performance of an auditory verbal memory: Between-subjects analysis by fMRI.** Brain Res Bull 2004, **64**:115-126.

55. Jabbi M, Swart M,Keysers C.: **Empathy for positive and negative emotions in the gustatory cortex.** Neuroimage 2007, **34**:1744-1753.

56. Jackson P L, Meltzoff A N,Decety J.: **How do we perceive the pain of others? A window into the neural processes involved in empathy.** Neuroimage 2005, **24**:771-779.

57. Jacobsen T, Schubotz R I, Hofel L,Yves von Cramon D.: **Brain correlates of aesthetic judgment of beauty.** Neuroimage 2006, **29**:276-285.

58. Johnson S H, Rotte M, Grafton S T, Hinrichs H, Gazzaniga M S,Heinze H J.: **Selective activation of a parietofrontal circuit during implicitly imagined prehension.** Neuroimage 2002, **17**:1693-1704.

59. Johnson-Frey S H, Newman-Norlund R,Grafton S T.: **A distributed left hemisphere network active during planning of everyday tool use skills.** Cerebral Cortex 2005, **15**:681-695.

60. Katzir T, Misra M,Poldrack R A.: **Imaging phonology without print: Assessing the neural correlates of phonemic awareness using fMRI.** Neuroimage 2005, **27**:106-115.

61. Keightley M L, Chiew K S, Winocur G,Grady C L.: **Age-related differences in brain activity underlying identification of emotional expressions in faces.** Social Cognitive and Affective Neuroscience 2007, **2**:292-302.

62. Kelley W M, Miezin F M, McDermott K B, Buckner R L, Raichle M E, Cohen N J, Ollinger J M, Akbudak E, Conturo T E, Snyder A Z,Petersen S E.: **Hemispheric specialization in human dorsal frontal cortex and medial temporal lobe for verbal and nonverbal memory encoding.** Neuron 1998, **20**:927-936.

63. Kemeny S, Ye F Q, Birn R M,Braun A R.: **Comparison of continuous overt speech fMRI using BOLD and arterial spin labeling.** Hum Brain Mapp 2005, **24**:173-183.

64. Kerns J G, Cohen J D, MacDonald III A W, Johnson M K, Stenger V A, Aizenstein H,Carter C S.: **Decreased conflict- and error-related activity in the anterior cingulate cortex in subjects with schizophrenia.** Am J Psychiatry 2005, **162**:1833-1839.

65. Kikyo H MY: **Temporal lobe activations of "feeling-of-knowing" induced by face-name associations.** Neuroimage 2004, **23**:1348-1357.

66. Kircher T T J, Brammer M J, Bullmore E T, Simmons A, Bartels M,David A S.: **The neural correlates of intentional and incidental self processing.** Neuropsychologia 2002, **40**:683-692.

67. Kitada R, Hashimoto T, Kochiyama T, Kito T, Okada T, Matsumura M, Lederman S J,Sadato N.: **Tactile estimation of the roughness of gratings yields a graded response in the human brain: An fMRI study.** Neuroimage 2005, **25**:90-100.

68. Knutson K M, Wood J N, Spampinato M V,Grafman J.: **Politics on the brain: An fMRI investigation.** Social Neuroscience 2006, **1**:25-40.

69. Koshino H, Carpenter P A, Keller T A,Just M A.: **Interactions between the dorsal and the ventral pathways in mental rotation: An fMRI study.** Cognitive, Affective, & Behavioral Neuroscience 2005, **5**:54-66.

70. Kringelbach M L,Rolls E T.: **Neural correlates of rapid reversal learning in a simple model of human social interaction.** Neuroimage 2003, **20**:1371-1383.

71. Kuhtz-Buschbeck J P, Mahnkopf C, Holzknecht C, Siebner H R, Ulmer S,Jansen O.: **Effector-independent representations of simple and complex imagined finger movements: A combined fMRI and TMS study.** Eur J Neurosci 2003, **18**:3375-3387.

72. Kumari V, Aasen I, Taylor P, Ffytche D H, Das M, Barkataki I, Goswami S, O'Connell P, Howlett M, Williams S C R,Sharma T S.: **Neural dysfunction and violence in schizophrenia: An fMRI investigation.** Schizophr Res 2006, **84**:144-164.

73. Kuo W J, Yeh T C, Duann J R, Wu Y T, Ho L T, Hung D L, Tzeng O J L,Hsieh J C.: **A left-lateralized network for reading Chinese words: a 3T fMRI study.** Neuroreport 2001, **12**:3997-4001.

74. Kuo W J, Yeh T C, Lee J R, Chen L F, Lee P L, Chen S S, Ho L T, Hung D L, Tzeng O J L,Hsieh J C.: **Orthographic and phonological processing of Chinese characters: An fMRI study.** Neuroimage 2004, **21**:1721-1731.

75. Lazeron R H C, Rombouts S A R B, de Sonneville L, Barkhof F,Scheltens P.: **A paced visual serial addition test for fMRI.** J Neurol Sci 2003, **213**:29-34.

76. Leung H C, Skudlarski P, Gatenby J C, Peterson B S,Gore J C.: **An event-related functional MRI study of the Stroop color word interference task.** Cerebral Cortex 2000, **10**:552-560.

77. Linden D E J, Bittner R A, Muckli L, Waltz J A, Kriegeskorte N, Goebel R, Singer W,Munk M H J.: **Cortical capacity constraints for visual working memory: Dissociation of fMRI load effects in a fronto-parietal network.** Neuroimage 2003, **20**:1518-1530.

78. Liu C, Zhang W T, Tang Y Y, Mai X Q, Chen H C, Tardif T,Luo Y J.: **The visual word form area: Evidence from an fMRI study of implicit processing of Chinese characters.** Neuroimage 2008, **40**:1350-1361.

79. Liu Y, Dunlap S, Fiez J,Perfetti C.: **Evidence for neural accommodation to a writing system following learning.** Hum Brain Mapp 2007, **28**:1223-1234.

80. Longcamp M, Anton J L, Roth M,Velay J L.: **Visual presentation of single letters activates a premotor area involved in writing.** Neuroimage 2003, **19**:1492-1500.

81. Luke K, Liu H L, Wai Y, Wan Y,Tan L H.: **Functional anatomy of syntactic and semantic processing in language comprehension.** Hum Brain Mapp 2002, **16**:133-145.

82. M. LK: **Cortical areas differentially involved in multiplication and subtraction: A functional magnetic resonance imaging study and correlation with a case of selective acalculia.** Ann Neurol 2000, **48**:657-661.

83. Malik S, McGlone F, Bedrossian D,Dagher A.: **Ghrelin modulates brain activity in areas that control appetitive behavior.** Cell Metabolism 2008, **7**:400-409.

84. Mattay V S, Callicott J H, Bertolino A, Santha A K S, Van Horn J D, Tallent K A, Frank J A,Weinberger D R.: **Hemispheric control of motor function: A whole brain echo planar fMRI study.** Psychiatry Res 1998, **83**:7-22.

85. Mead L A, Mayer A R, Bobholz J A, Woodley S J, Cunningham J M, Hammeke T A,Rao S M.: **Neural basis of the Stroop interference task: response competition or selective attention?** Journal of the International Neuropsychological Society 2002, **8**:735-742.

86. Meister I LM: **No language-specific activation during linguistic processing of observed actions.** PLoS ONE 2007, **2**:891-891.

87. Milham M P, Banich M T, Webb A G, Barad V, Cohen N J, Wszalek T M,Kramer A F.: **The relative involvement of anterior cingulate and prefrontal cortex in attentional control depends on nature of conflict.** Cognitive Brain Research 2001, **12**:467-473.

88. Monchi O, Petrides M, Doyon J, Postuma R B, Worsley K J,Dagher A.: **Neural bases of set-shifting deficits in Parkinson's disease.** Journal of Neuroscience 2004, **24**:702-710.

89. Montaldi D, Spencer T J, Roberts N,Mayes A R.: **The neural system that mediates familiarity memory.** Hippocampus 2006, **16**:504-520.

90. Moriguchi Y, Decety J, Ohnishi T, Maeda M, Mori T, Nemoto K, Matsuda H,Komaki G.: **Empathy and judging other's pain: An fMRI study of alexithymia.** Cerebral Cortex 2007, **17**:2223-2234.

91. Nagahama Y, Okada T, Katsumi Y, Hayashi T, Yamauchi H, Oyanagi C, Konishi J, Fukuyama H,Shibasaki H.: **Dissociable mechanisms of attentional control within the human prefrontal cortex.** Cerebral Cortex 2001, **11**:85-92.

92. Najib A, Lorberbaum J P, Kose S, Bohning D E,George M S.: **Regional brain activity in women grieving a romantic relationship breakup.** Am J Psychiatry 2004, **161**:2245-2256.

93. Nakamura K, Honda M, Okada T, Hanakawa T, Toma K, Fukuyama H, Konishi J,Shibasaki H.: **Participation of the left posterior inferior temporal cortex in writing and mental recall of kanji orthography: A functional MRI study.** Brain 2000, **123**:954-967.

94. Ng V W K, Bullmore E T, de Zubicaray G I, Cooper A, Suckling J,Williams S C R.: **Identifying rate-limiting nodes in large-scale cortical networks for visuospatial processing: An illustration using fMRI.** J Cogn Neurosci 2001, **13**:537-545.

95. Parsons M W, Harrington D L,Rao S M.: **Distinct neural systems underlie learning visuomotor and spatial representations of motor skills.** Hum Brain Mapp 2005, **24**:229-247.

96. Peterson B S, Skudlarski P, Gatenby J C, Zhang H, Anderson A W,Gore J C.: **An fMRI study of Stroop word-color interference: evidence for cingulate subregions subserving multiple distributed attentional systems.** Biol Psychiatry 1999, **45**:1237-1258.

97. Pihlajamaki M, Tanila H, Hanninen T, Kononen M, Mikkonen M, Jalkanen V, Partanen K, Aronen H J,Soininen H.: **Encoding of novel picture pairs activates the perirhinal cortex: An fMRI study.** Hippocampus 2003, **13**:67-80.

98. Pinel P, Dehaene S, Riviere D,LeBihan D.: **Modulation of parietal activation by semantic distance in a number comparison task.** Neuroimage 2001, **14**:1013-1026.

99. Ponseti J, Bosinski H A, Wolff S, Peller M, Jansen O, Mehdorn H M, Buchel C,Siebner H R.: **A functional endophenotype for sexual orientation in humans.** Neuroimage 2006, **33**:825-833.

100. Ragland J D, Gur R C, Valdez J N, Loughead J, Elliot M A, Kohler C, Kanes S, Siegel S J, Moelter S T,Gur R E.: **Levels-of-processing effect on frontotemporal function in schizophrenia during word encoding and recognition.** Am J Psychiatry 2005, **162**:1840-1848.

101. Ragland J D, Turetsky B I, Gur R C, Gunning-Dixon F, Turner T, Schroeder L, Chan R,Gur R E.: **Working memory for complex figures: An fMRI comparison of letter and fractal n-back tasks.** Neuropsychology 2002, **16**:370-379.

102. Ragland J D, Valdez J N, Loughead J, Gur R C,Gur R E.: **Functional magnetic resonance imaging of internal source monitoring in schizophrenia: Recognition with and without recollection.** Schizophr Res 2006, **87**:160-171.

103. Ranganath C, Yonelinas A P, Cohen M X, Dy C J, Tom S M,D'Esposito M.: **Dissociable correlates of recollection and familiarity within the medial temporal lobes.** Neuropsychologia 2003, **42**:2-13.

104. Ricciardi E, Bonino D, Gentili C, Sani L, Pietrini P,Vecchi T.: **Neural correlates of spatial working memory in humans: A functional magnetic resonance imaging study comparing visual and tactile processes.** Neuroscience 2006, **139**:339-349.

105. Ruff C C, Woodward T S, Laurens K R,Liddle P F.: **The role of the anterior cingulate cortex in conflict processing: evidence from reverse Stroop interference.** Neuroimage 2001, **14**:1150-1158.

106. Rypma B, Prabhakaran V, Desmond J E, Glover G H,Gabrieli J D E.: **Load-dependent roles of frontal brain regions in the maintenance of working memory.** Neuroimage 1999, **9**:216-226.

107. Sanchez-Carrion R, Gomez P V, Junque C, Fernandez-Espejo D, Falcon C, Bargallo N, Roig-Rovira T, Ensenat-Cantallops A,Bernabeu M.: **Frontal hypoactivation on functional magnetic resonance imaging in working memory after severe diffuse traumatic brain injury.** J Neurotrauma 2008, **25**:479-494.

108. Schlosser R, Hutchinson M, Joseffer S, Rusinek H, Saarimaki A, Stevenson J, Dewey S L,Brodie J D.: **Functional magnetic resonance imaging of human brain activity in a verbal fluency task.** J Neurol Neurosurg Psychiatr 1998, **64**:492-498.

109. Schoning S, Engelien A, Kugel H, Schafer S, Schiffbauer H, Zwitserlood P, Pletziger E, Beizai P, Kersting A, Ohrmann P, Greb R R, Lehmann W, Heindel W, Arolt V,Konrad C.: **Functional anatomy of visuo-spatial working memory during mental rotation is influenced by sex, menstrual cycle, and sex steroid hormones.** Neuropsychologia 2007, **45**:3203-3214.

110. Seurinck R, Vingerhoets G, de Lange F P,Achten E.: **Does egocentric mental rotation elicit sex differences?** Neuroimage 2004, **23**:1440-1449.

111. Shulman G L, Ollinger J M, Akbudak E, Conturo T E, Snyder A Z, Petersen S E,Corbetta M.: **Areas involved in encoding and applying directional expectations to moving objects.** Journal of Neuroscience 1999, **19**:9480-9496.

112. Simmons W K, Hamann S B, Harenski C L, Hu X,Barsalou L W.: **fMRI evidence for word association and situated simulation in conceptual processing.** Journal of Physiology - Paris 2008, **102**:106-119.

113. Siok W T, Jin Z, Fletcher P C,Tan L H.: **Distinct brain regions associated with syllable and phoneme.** Hum Brain Mapp 2003, **18**:201-207.

114. Siok W T, Perfetti C A, Jin Z,Tan L H.: **Biological abnormality of impaired reading is constrained by culture.** Nature 2004, **431**:71-76.

115. Small G W, Moody T D, Siddarth P,Bookheimer S Y.: **Your brain on Google: Patterns of cerebral activation during internet searching.** American Journal of Geriatric Psychiatry 2009, **17**:116-126.

116. Smith C D, Andersen A H, Kryscio R J, Schmitt F A, Kindy M S, Blonder L X,Avison M J.: **Differences in functional magnetic resonance imaging activation by category in a visual confrontation naming task.** Journal of Neuroimaging 2001, **11**:165-170.

117. Specht K RJ: **Functional segregation of the temporal lobes into highly differentiated subsystems for auditory perception: An auditory rapid event-related fMRI-task.** Neuroimage 2003, **20**:1944-1954.

118. Stanescu-Cosson R, Pinel P, Van De Moortele P F, LeBihan D, Cohen L G,Dehaene S.: **Understanding dissociations in dyscalculia: A brain imaging study of the impact of number size on the cerebral networks for exact and approximate calculation.** Brain 2000, **123**:2240-2255.

119. Staudt M, Lidzba K, Grodd W, Wildgruber D, Erb M,Krageloh-Mann I.: **Right-hemispheric organization of language following early left-sided brain lesions: Functional MRI topography.** Neuroimage 2002, **16**:954-967.

120. Summerfield J J, Hassabis D,Maguire E A.: **Cortical midline involvement in autobiographical memory.** Neuroimage 2009, **44**:1188-1200.

121. Szameitat A J, Schubert T, Muller K,Yves von Cramon D.: **Localization of executive functions in dual-task performance with fMRI.** J Cogn Neurosci 2002, **14**:1184-1199.

122. Toyomura A, Koyama S, Miyamaoto T, Terao A, Omori T, Murohashi H,Kuriki S.: **Neural correlates of auditory feedback control in human.** Neuroscience 2007, **146**:499-503.

123. Uher R, Treasure J, Heining M, Brammer M J,Campbell I C.: **Cerebral processing of food-related stimuli: Effects of fasting and gender.** Behav Brain Res 2006, **169**:111-119.

124. Uncapher M R, Otten L J,Rugg M D.: **Episodic encoding is more than the sum of its parts: An fMRI investigation of multifeatural contextual encoding.** Neuron 2006, **52**:547-556.

125. van Dijk P,Backes W H.: **Brain activity during auditory backward and simultaneous masking tasks.** Hear Res 2003, **181**:8-14.

126. van Turennout M, Bielamowicz L,Martin A.: **Modulation of neural activity during object naming: Effects of time and practice.** Cerebral Cortex 2003, **13**:381-391.

127. Vannini P, Lehmann C, Dierks T, Jann K, Viitanen M, Wahlund L O,Almkvist O.: **Failure to modulate neural response to increased task demand in mild Alzheimer's disease: fMRI study of visuospatial processing.** Neurobiol Dis 2008, **31**:287-297.

128. Vingerhoets G, Van Borsel J, Tesink C, van den Noort M, Deblaere K, Seurinck R, Vandemaele P,Achten E.: **Multilingualism: An fMRI study.** Neuroimage 2003, **20**:2181-2196.

129. Volle E, Pochon J B, Lehericy S, Pillon B, Dubois B,Levy R.: **Specific cerebral networks for maintenance and response organization within working memory as evidenced by the 'double delay/double response' paradigm.** Cerebral Cortex 2005, **15**:1064-1074.

130. Wang A T, Dapretto M, Hariri A R, Sigman M,Bookheimer S Y.: **Neural correlates of facial affect processing in children and adolescents with autism spectrum disorder.** J Am Acad Child Adolesc Psychiatry 2004, **43**:481-490.

131. Weiss E M, Siedentopf C M, Golaszewski S, Mottaghy F M, Hofer A, Kremser C, Felber S M,Fleischhacker W W.: **Brain activation patterns during a selective attention test - a functional MRI study in healthy volunteers and unmedicated patients during an acute episode of schizophrenia.** Psychiatry Res 2007, **154**:31-40.

132. Whalen P J, Bush G, McNally R J,Wilhelm S.: **The emotional counting Stroop paradigm: A functional magnetic resonance imaging probe of the anterior cingulate affective division.** Biol Psychiatry 1998, **44**:1219-1228.

133. Wildgruber D, Riecker A, Hertrich I, Erb M, Grodd W, Ethofer T,Ackermann H.: **Identification of emotional intonation evaluated by fMRI.** Neuroimage 2005, **24**:1233-1241.

134. Williams L M, Phillips M L, Brammer M J, Skerrett D, Lagopoulos J, Rennie C, Bahramali H, Olivieri G, David A S, Peduto A,Gordon E.: **Arousal dissociates amygdala and hippocampal fear responses: evidence from simultaneous fMRI and skin conductance recording.** Neuroimage 2001, **14**:1070-1079.

135. Wong P C M, Uppunda A K, Parrish T B,Dhar S.: **Cortical mechanisms of speech perception in noise.** Journal of Speech, Language, and Hearing Research 2008, **51**:1026-1041.

136. Wraga M, Shephard J M, Church J A, Inati S,Kosslyn S M.: **Imagined rotations of self versus objects: An fMRI study.** Neuropsychologia 2005, **43**:1351-1361.

137. Wunderlich K, Rangel A,O'Doherty J P.: **Neural computations underlying action-based decisionmaking in the human brain.** Proceedings of the National Academy of Sciences 2009, **106**:17199-17204.

138. Xu B, Grafman J, Gaillard W D, Spanaki M, Ishii K, Balsamo L M, Makale M,Theodore W H.: **Neuroimaging reveals automatic speech coding during perception of written word meaning.** Neuroimage 2002, **17**:859-870.

139. Yoo S S, Choi B G, Juh R H, Park J M, Pae C U, Kim J J, Lee S J, Lee C, Paik I H,Lee C U.: **Working memory processing of facial images in schizophrenia: fMRI investigation.** Int J Neurosci 2005, **115**:351-366.

B – 111 articles initially identified for the right IFJ

1. Abel K M, Allin M P G, Kucharska-Pietura K, Andrew C M, Williams S C R, David A S,Phillips M L.: **Ketamine and fMRI BOLD signal: distinguishing between effects mediated by change in blood flow versus change in cognitive state.** Hum Brain Mapp 2003, **18**:135-145.

2. Achim A M LM: **Neural correlates of memory for items and for associations: An event-related functional magnetic resonance imaging study.** J Cogn Neurosci 2005, **17**:652-667.

3. Addis D R, Wong A T,Schacter D L.: **Remembering the past and imagining the future: Common and distinct neural substrates during event construction and elaboration.** Neuropsychologia 2007, **45**:1363-1377.

4. Ahrens K, Liu H L, Lee C Y, Gong S P, Fang S Y,Hsu Y Y.: **Functional MRI of conventional and anomalous metaphors in metaphors in Mandarin Chinese.** Brain Lang 2007, **100**:163-171.

5. Astafiev S V, Shulman G L, Stanley C M, Snyder A Z, Van Essen D C,Corbetta M.: **Functional organization of human intraparietal and frontal cortex for attending, looking, and pointing.** Journal of Neuroscience 2003, **23**:4689-4699.

6. Baumgartner T, Lutz K, Schmidt C F,Jancke L.: **The emotional power of music: How music enhances the feeling of affective pictures.** Brain Res 2006, **1075**:151-164.

7. Becerra L R, Breiter H C, Wise R, Gonzalez R G,Borsook D.: **Reward circuitry activation by noxious thermal stimuli.** Neuron 2001, **32**:927-946.

8. Binder J R, McKiernan K A, Parsons M E, Westbury C F, Possing E T, Kaufman J N,Buchanan L.: **Neural correlates of lexical access during visual word recognition.** J Cogn Neurosci 2003, **15**:372-393.

9. Bohland J W,Guenther F H.: **An fMRI investigation of syllable sequence production.** Neuroimage 2006, **32**:821-841.

10. Brass M,Yves von Cramon D.: **The role of the frontal cortex in task preparation.** Cerebral Cortex 2002, **12**:908-914.

11. Braver T S, Barch D M, Gray J R, Molfese D L,Snyder A Z.: **Anterior cingulate cortex and response conflict: effects of frequency, inhibition and errors.** Cerebral Cortex 2001, **11**:825-836.

12. Braver T S, Cohen J D, Nystrom L E, Jonides J, Smith E E,Noll D C.: **A parametric study of prefrontal cortex involvement in human working memory.** Neuroimage 1997, **5**:49-62.

13. Buchsbaum B R, Olsen R K, Koch P F, Kohn P, Kippenhan J S,Berman K F.: **Reading, hearing, and the planum temporale.** Neuroimage 2005, **24**:444-454.

14. Budhani S, Marsh A A, Pine D S,Blair R J R.: **Neural correlates of response reversal: Considering acquisition.** Neuroimage 2007, **34**:1754-1765.

15. Callan D E, Tsytsarev V, Hanakawa T, Callan A M, Katsuhara M, Fukuyama H,Turner R S.: **Song and speech: Brain regions involved with perception and covert production.** Neuroimage 2006, **31**:1327-1342.

16. Camchong J, Dyckman K A, Chapman C E, Yanasak N E,McDowell J E.: **Basal ganglia-thalamocortical circuitry disruptions in schizophrenia during delayed response tasks.** Biol Psychiatry 2006, **60**:235-241.

17. Cansino S, Maquet P, Dolan R J,Rugg M D.: **Brain activity underlying encoding and retrieval of source memory.** Cerebral Cortex 2002, **12**:1048-1056.

18. Carr L, Iacoboni M, Dubeau M C, Mazziotta J C,Lenzi G L.: **Neural mechanisms of empathy in humans: A relay from neural systems for imitation to limbic areas.** Proceedings of the National Academy of Sciences 2003, **100**:5497-5502.

19. Chikazoe J, Jimura K, Asari T, Yamashita K, Morimoto H, Hirose S, Miyashita Y,Konishi S.: **Functional dissociation in right inferior frontal cortex during performance of go/no-go task.** Cerebral Cortex 2009, **19**:146-152.

20. Chikazoe J, Jimura K, Hirose S, Yamashita K, Miyashita Y,Konishi S.: **Preparation to inhibit a response complements response inhibition during performance of a stop-signal task.** Journal of Neuroscience 2009, **29**:15870-15877.

21. Chochon F, Cohen L G, Van De Moortele P F,Dehaene S.: **Differential contributions of the left and right inferior parietal lobules to number processing.** J Cogn Neurosci 1999, **11**:617-630.

22. Cohen L G, Lehericy S, Chochon F, Lemer C, Rivaud S,Dehaene S.: **Language-specific tuning of visual cortex? Functional properties of the Visual Word Form Area.** Brain 2002, **125**:1054-1069.

23. Connolly J D, Goodale M A, DeSouza J F, Menon R S,Vilis T.: **A comparison of frontoparietal fMRI activation during anti-saccades and anti-pointing.** J Neurophysiol 2000, **84**:1645-1655.

24. de Leeuw R, Davis C E, Albuquerque R, Carlson C R,Andersen A H.: **Brain activity during stimulation of the trigeminal nerve with noxious heat.** Oral Surg Oral Med Oral Pathol Oral Radiol Endod 2006, **102**:750-757.

25. Delgado M R, Nystrom L E, Fissell C, Noll D C,Fiez J A.: **Tracking the hemodynamic responses to reward and punishment in the striatum.** J Neurophysiol 2000, **84**:3072-3077.

26. Dichter G S BA: **Social stimuli interfere with cognitive control in autism.** Neuroimage 2007, **35**:1219-1230.

27. Dolcos F, LaBar K S,Cabeza R.: **Dissociable effects of arousal and valence on prefrontal activity indexing emotional evaluation and subsequent memory: An event-related fMRI study.** Neuroimage 2004, **23**:64-74.

28. Drobyshevsky A, Baumann S B,Schneider W.: **A rapid fMRI task battery for mapping of visual, motor, cognitive, and emotional function.** Neuroimage 2006, **31**:732-744.

29. Ehrsson H H, Fagergren A, Jonsson T, Westling G, Johansson R S,Forssberg H.: **Cortical activity in precision- versus power-grip task: An fMRI study.** J Neurophysiol 2000, **83**:528-536.

30. Fan J, Flombaum J I, McCandliss B D, Thomas K M,Posner M I.: **Cognitive and brain consequences of conflict.** Neuroimage 2003, **18**:42-57.

31. Forstmann B U, van den Wildenberg W P M,Ridderinkhof K R.: **Neural mechanisms, temporal dynamics, and individual differences in interference control.** J Cogn Neurosci 2008, **20**:1854-1865.

32. Frangou S, Kington J, Raymont V,Shergill S S.: **Examining ventral and dorsal prefrontal function in bipolar disorder: A functional magnetic resonance imaging study.** European Psychiatry 2008, **23**:300-308.

33. Garavan H, Ross T J, Li S J,Stein E A.: **A parametric manipulation of central executive functioning.** Cerebral Cortex 2000, **10**:585-592.

34. Gowen E MRC: **Differentiation between external and internal cuing: An fMRI study comparing tracing with drawing.** Neuroimage 2007, **36**:396-410.

35. Gruber O,Yves von Cramon D.: **The functional neuroanatomy of human working memory revisited: Evidence from 3-T fMRI studies using classical domain-specific interference tasks.** Neuroimage 2003, **19**:797-809.

36. Gur R C, Turetsky B I, Loughead J, Waxman J, Snyder W, Ragland J D, Elliot M A, Bilker W B, Arnold S E,Gur R E.: **Hemodynamic response in neural circuitries for detection of visual target and novelty: An event-related fMRI study.** Hum Brain Mapp 2007, **28**:263-274.

37. Hanakawa T, Dimyan M A,Hallett M.: **Motor planning, imagery, and execution in the distributed motor network: A time-course study with functional MRI.** Cerebral Cortex 2008, **18**:2775-2788.

38. Hart H C, Palmer A R,Hall D A.: **Different areas of human non-primary auditory cortex are activated by sounds with spatial and non spatial resolution.** Hum Brain Mapp 2004, **21**:178-190.

39. Helmchen C, Mohr C, Roehl M, Bingel U, Lorenz J,Buchel C.: **Common neural systems for contact heat and laser pain stimulation reveal higher-level pain processing.** Hum Brain Mapp 2008, **29**:1080-1091.

40. Husain F T, Fromm S J, Pursley R H, Hosey L A, Braun A R,Horwitz B.: **Neural bases of categorization of simple speech and nonspeech sounds.** Hum Brain Mapp 2006, **27**:636-651.

41. Husain F T, McKinney C M,Horwitz B.: **Frontal cortex functional connectivity changes during sound categorization.** Neuroreport 2006, **17**:617-621.

42. Ilg R, Vogeley K, Goschke T, Bolte A, Shah J N, Poppel E,Fink G R.: **Neural processes underlying intuitive coherence judgments as revealed by fMRI on a semantic judgment task.** Neuroimage 2007, **38**:228-238.

43. Ino T, Doi T, Kimura T, Ito J,Fukuyama H.: **Neural substrates of the performance of an auditory verbal memory: Between-subjects analysis by fMRI.** Brain Res Bull 2004, **64**:115-126.

44. Ino T, Nakai R, Azuma T, Tokumoto K, Usami K,Kimura T.: **An FMRI study of word reading and colour recognition in different quadrant fields.** Open Neuroimaging Journal 2008, **2**:56-64.

45. Iseki K, Hanakawa T, Shinozaki J, Nankaku M,Fukuyama H.: **Neural mechanisms involved in mental imagery and observation of gait.** Neuroimage 2008, **41**:1021-1031.

46. Jordan K, Heinze H J, Lutz K, Kanowski M,Jancke L.: **Cortical activations during the mental rotation of different visual objects.** Neuroimage 2001, **13**:143-152.

47. Jordan K, Wustenberg T, Heinze H J, Peters M,Jancke L.: **Women and men exhibit different cortical activation patterns during mental rotation tasks.** Neuropsychologia 2002, **40**:2397-2408.

48. Just M A, Newman S D, Keller T A, McEleney A,Carpenter P A.: **Imagery in sentence comprehension: An fMRI study.** Neuroimage 2004, **21**:112-124.

49. Kato J, Ide H, Kabashima I, Kadota H, Takano K,Kansaku K.: **Neural correlates of attitude change following positive and negative advertisements.** Behav Neurosci 2009, **3**:6-6.

50. Kelley W M, Macrae C N, Wyland C L, Caglar S, Inati S,Heatherton T F.: **Finding the self? An event-related fMRI study.** J Cogn Neurosci 2002, **14**:785-794.

51. Kerns J G, Cohen J D, MacDonald III A W, Johnson M K, Stenger V A, Aizenstein H,Carter C S.: **Decreased conflict- and error-related activity in the anterior cingulate cortex in subjects with schizophrenia.** Am J Psychiatry 2005, **162**:1833-1839.

52. Knutson B, Wimmer G E, Kuhnen C M,Winkielman P.: **Nucleus accumbens activation mediates the influence of reward cues on financial risk taking.** Neuroreport 2008, **19**:509-513.

53. Konen C S, Kleiser R, Wittsack H J, Bremmer F,Seitz R J.: **The encoding of saccadic eye movements within human posterior parietal cortex.** Neuroimage 2004, **22**:304-314.

54. Konishi S, Nakajima K, Uchida I, Sekihara K,Miyashita Y.: **No-go dominant brain activity in human inferior prefrontal cortex revealed by functional magnetic resonance imaging.** Eur J Neurosci 1998, **10**:1209-1213.

55. Kroger J K, Sabb F W, Fales C L, Bookheimer S Y, Cohen M S,Holyoak K J.: **Recruitment of anterior dorsolateral prefrontal cortex in human reasoning: a parametric study of relational complexity.** Cerebral Cortex 2002, **12**:477-485.

56. Kuhtz-Buschbeck J P, Mahnkopf C, Holzknecht C, Siebner H R, Ulmer S,Jansen O.: **Effector-independent representations of simple and complex imagined finger movements: A combined fMRI and TMS study.** Eur J Neurosci 2003, **18**:3375-3387.

57. Kumari V, Aasen I, Taylor P, Ffytche D H, Das M, Barkataki I, Goswami S, O'Connell P, Howlett M, Williams S C R,Sharma T S.: **Neural dysfunction and violence in schizophrenia: An fMRI investigation.** Schizophr Res 2006, **84**:144-164.

58. LaBar K S, Gitelman D R, Parrish T B,Mesulam M M.: **Neuroanatomic overlap of working memory and spatial attention networks: A functional MRI comparison within subjects.** Neuroimage 1999, **10**:695-704.

59. Lanius R A, Williamson P C, Densmore M, Boksman K, Neufeld R W, Gati J S,Menon R S.: **The nature of traumatic memories: A 4-T FMRI functional connectivity analysis.** Am J Psychiatry 2004, **161**:36-44.

60. Lee T W, Josephs O, Dolan R J,Critchley H D.: **Imitating expressions: Emotion-specific neural substrates in facial mimicry.** Social Cognitive and Affective Neuroscience 2006, **1**:122-135.

61. Leslie K R, Johnson-Frey S H,Grafton S T.: **Functional imaging of face and hand imitation: Towards a motor theory of empathy.** Neuroimage 2004, **21**:601-607.

62. Leung H C, Skudlarski P, Gatenby J C, Peterson B S,Gore J C.: **An event-related functional MRI study of the Stroop color word interference task.** Cerebral Cortex 2000, **10**:552-560.

63. Mainero C, Caramia F, Pozzilli C, Pisani A, Pestalozza I, Borriello G, Bozzao L,Pantano P.: **fMRI evidence of brain reorganization during attention and memory tasks in multiple sclerosis.** Neuroimage 2004, **21**:858-867.

64. Malik S, McGlone F, Bedrossian D,Dagher A.: **Ghrelin modulates brain activity in areas that control appetitive behavior.** Cell Metabolism 2008, **7**:400-409.

65. Martin R E, Goodyear B G, Gati J S,Menon R S.: **Cerebral cortical representation of automatic and volitional swallowing in humans.** J Neurophysiol 2001, **85**:938-950.

66. Mattay V S, Callicott J H, Bertolino A, Santha A K S, Van Horn J D, Tallent K A, Frank J A,Weinberger D R.: **Hemispheric control of motor function: A whole brain echo planar fMRI study.** Psychiatry Res 1998, **83**:7-22.

67. McNab F, Leroux G, Strand F, Thorell L, Bergman S,Klingberg T.: **Common and unique components of inhibition and working memory: An fMRI, within-subjects investigation.** Neuropsychologia 2008, **46**:2668-2682.

68. Meisenzahl E M, Scheuerecker J, Zipse M, Ufer S, Wiesmann M, Frodl T, Koutsouleris N, Zetzsche T, Schmitt G, Riedel M, Spellmann I, Dehning S, Linn J, Bruckmann H,Moller H J.: **Effects of treatment with the atypical neuroleptic quetiapine on working memory function: A functional MRI follow-up investigation.** Eur Arch Psychiatry Clin Neurosci 2006, **256**:522-531.

69. Mendrek A, Laurens K R, Kiehl K A, Ngan E T C, Stip E,Liddle P F.: **Changes in distributed neural circuitry function in patients with first-episode schizophrenia.** British Journal of Psychiatry 2004, **185**:205-214.

70. Milham M P, Banich M T,Barad V.: **Competition for priority in processing increases prefrontal cortex's involvement in top-down control: An event-related fMRI study of the stroop task.** Cognitive Brain Research 2003, **17**:212-222.

71. Mochizuki-Kawai H, Tsukiura T, Mochizuki S,Kawamura M.: **Learning-related changes of brain activation in the visual ventral stream: An fMRI study of mirror reading skill.** Brain Res 2006, **1122**:154-160.

72. Monchi O, Petrides M, Petre V, Worsley K J,Dagher A.: **Wisconsin Card Sorting revisited: Distinct neural circuits participating in different stages of the task identified by event-related functional magnetic resonance imaging.** Journal of Neuroscience 2001, **21**:7733-7741.

73. Moriguchi Y, Decety J, Ohnishi T, Maeda M, Mori T, Nemoto K, Matsuda H,Komaki G.: **Empathy and judging other's pain: An fMRI study of alexithymia.** Cerebral Cortex 2007, **17**:2223-2234.

74. Niddam D M, Yeh T C, Wu Y T, Lee P L, Ho L T, Arendt-Nielsen L, Chen A C N,Hsieh J C.: **Event-related functional MRI study on central representation of acute muscle pain induced by electrical stimulation.** Neuroimage 2002, **17**:1437-1450.

75. Noriuchi M, Kikuchi Y,Senoo A.: **The functional neuroanatomy of maternal love: Mother's response to infant's attachment behaviors.** Biol Psychiatry 2008, **63**:415-423.

76. Notebaert K, Pesenti M,Reynvoet B.: **The neural origin of the priming distance effect: Distance-dependent recovery of parietal activation using symbolic magnitudes.** Hum Brain Mapp 2010, **31**:669-677.

77. Olson I R, Gatenby J C,Gore J C.: **A comparison of bound and unbound audio-visual information processing in the human cerebral cortex.** Cognitive Brain Research 2002, **14**:129-138.

78. Peelen M V, Heslenfeld D J,Theeuwes J.: **Endogenous and exogenous attention shifts are mediated by the same large-scale neural network.** Neuroimage 2004, **22**:822-830.

79. Peng D L, Xu D, Jin Z, Luo Q, Ding G S, Perry C, Zhang L,Liu Y.: **Neural basis of the non-attentional processing of briefly presented words.** Hum Brain Mapp 2003, **18**:215-221.

80. Peterson B S, Skudlarski P, Gatenby J C, Zhang H, Anderson A W,Gore J C.: **An fMRI study of Stroop word-color interference: evidence for cingulate subregions subserving multiple distributed attentional systems.** Biol Psychiatry 1999, **45**:1237-1258.

81. Pollmann S, Dove A, Yves von Cramon D,Wiggins C J.: **Event-related fMRI: Comparison of conditions with varying BOLD overlap.** Hum Brain Mapp 2000, **9**:26-37.

82. Ponseti J, Bosinski H A, Wolff S, Peller M, Jansen O, Mehdorn H M, Buchel C,Siebner H R.: **A functional endophenotype for sexual orientation in humans.** Neuroimage 2006, **33**:825-833.

83. Potenza M N, Leung H C, Blumberg H P, Peterson B S, Fulbright R K, Lacadie C M, Skudlarski P,Gore J C.: **An fMRI Stroop task study of ventromedial prefrontal cortical function in pathological gamblers.** Am J Psychiatry 2003, **160**:1990-1994.

84. Quintana J, Wong T, Ortiz-Portillo E, Kovalik E, Davidson T, Marder S R,Mazziotta J C.: **Prefrontal-posterior parietal networks in schizophrenia: Primary dysfunctions and secondary compensations.** Biol Psychiatry 2003, **53**:12-24.

85. Ragland J D, Gur R C, Valdez J N, Loughead J, Elliot M A, Kohler C, Kanes S, Siegel S J, Moelter S T,Gur R E.: **Levels-of-processing effect on frontotemporal function in schizophrenia during word encoding and recognition.** Am J Psychiatry 2005, **162**:1840-1848.

86. Ricciardi E, Bonino D, Gentili C, Sani L, Pietrini P,Vecchi T.: **Neural correlates of spatial working memory in humans: A functional magnetic resonance imaging study comparing visual and tactile processes.** Neuroscience 2006, **139**:339-349.

87. Rossell S L, Bullmore E T, Williams S C R,David A S.: **Sex differences in functional brain activation during a lexical visual field task.** Brain Lang 2002, **80**:97-105.

88. Rubia K, Russell T, Overmeyer S, Brammer M J, Bullmore E T, Sharma T S, Simmons A, Williams S C R, Giampietro V, Andrew C M,Taylor E.: **Mapping motor inhibition: Conjunctive brain activations across different versions of go/no-go and stop tasks.** Neuroimage 2001, **13**:250-261.

89. Sacco K, Cauda F, Cerliani L, Mate D, Duca S,Geminiani G C.: **Motor imagery of walking following training in locomotor attention: The effect of "the tango lesson".** Neuroimage 2006, **32**:1441-1449.

90. Sanchez-Carrion R, Gomez P V, Junque C, Fernandez-Espejo D, Falcon C, Bargallo N, Roig-Rovira T, Ensenat-Cantallops A,Bernabeu M.: **Frontal hypoactivation on functional magnetic resonance imaging in working memory after severe diffuse traumatic brain injury.** J Neurotrauma 2008, **25**:479-494.

91. Schnell K HSC: **Effects of dialectic-behavioral-therapy on the neural correlates of affective hyperarousal in borderline personality disorder.** J Psychiatr Res 2007, **41**:837-847.

92. Shergill S S, Bullmore E T, Brammer M J, Williams S C R, Murray R M,McGuire P K.: **A functional study of auditory verbal imagery.** Psychol Med 2001, **31**:241-253.

93. Shikata E, Hamzei F, Glauche V, Koch M, Weiller C, Binkofski F,Buchel C.: **Functional properties and interaction of the anterior and posterior intraparietal areas in humans.** Eur J Neurosci 2003, **17**:1105-1110.

94. Shulman G L, Ollinger J M, Akbudak E, Conturo T E, Snyder A Z, Petersen S E,Corbetta M.: **Areas involved in encoding and applying directional expectations to moving objects.** Journal of Neuroscience 1999, **19**:9480-9496.

95. Small G W, Moody T D, Siddarth P,Bookheimer S Y.: **Your brain on Google: Patterns of cerebral activation during internet searching.** American Journal of Geriatric Psychiatry 2009, **17**:116-126.

96. Sperling R A, Bates J F, Cocchiarella A J, Schacter D L, Rosen B R,Albert M S.: **Encoding novel face-name associations: A functional MRI study.** Hum Brain Mapp 2001, **14**:129-139.

97. Stanescu-Cosson R, Pinel P, Van De Moortele P F, LeBihan D, Cohen L G,Dehaene S.: **Understanding dissociations in dyscalculia: A brain imaging study of the impact of number size on the cerebral networks for exact and approximate calculation.** Brain 2000, **123**:2240-2255.

98. Stark R, Zimmermann M, Kagerer S, Schienle A, Walter B, Weygandt M,Vaitl D.: **Hemodynamic brain correlates of disgust and fear ratings.** Neuroimage 2007, **37**:663-673.

99. Stern C E, Owen A M, Tracey I, Look R B, Rosen B R,Petrides M.: **Activity in ventrolateral and mid-dorsolateral prefrontal cortex during nonspatial visual working memory processing: Evidence from functional magnetic resonance imaging.** Neuroimage 2000, **11**:392-399.

100. Strathearn L, Li J, Fonagy P,Montague R.: **What's in a smile? maternal brain responses to infant facial cues.** Pediatrics 2008, **122**:40-51.

101. Straube T, Preissler S, Lipka J, Hewig J, Mentzel H J,Miltner W H R.: **Neural representation of anxiety and personality during exposure to anxiety-provoking and neutral scenes from scary movies.** Hum Brain Mapp 2010, **31**:36-47.

102. Tan L H, Feng C, Fox P T,Gao J H.: **An fMRI study with written Chinese.** Neuroreport 2001, **12**:83-88.

103. Tan L H, Lui H L, Perfetti C A, Spinks J A, Fox P T,Gao J H.: **The neural system underlying Chinese logograph reading.** Neuroimage 2001, **13**:836-846.

104. Tan L H, Spinks J A, Gao J H, Liu H L, Perfetti C A, Xiong J, Stofer K A, Pu Y, Liu Y,Fox P T.: **Brain activation in the processing of Chinese characters and words: A functional MRI study.** Hum Brain Mapp 2000, **10**:16-27.

105. Toyomura A, Koyama S, Miyamaoto T, Terao A, Omori T, Murohashi H,Kuriki S.: **Neural correlates of auditory feedback control in human.** Neuroscience 2007, **146**:499-503.

106. Ullsperger M,Yves von Cramon D.: **Subprocesses of performance monitoring: A dissociation of error processing and response competition revealed by event-related fMRI and ERPs.** Neuroimage 2001, **14**:1387-1401.

107. Uncapher M R,Rugg M D.: **Encoding and the durability of episodic memory: A functional magnetic resonance imaging study.** Journal of Neuroscience 2005, **25**:7260-7267.

108. Vannini P, Lehmann C, Dierks T, Jann K, Viitanen M, Wahlund L O,Almkvist O.: **Failure to modulate neural response to increased task demand in mild Alzheimer's disease: fMRI study of visuospatial processing.** Neurobiol Dis 2008, **31**:287-297.

109. Wagner A D, Pare-Blagoev E J, Clark J,Poldrack R A.: **Recovering meaning: Left prefrontal cortex guides controlled semantic retrieval.** Neuron 2001, **31**:329-338.

110. Wang A T, Dapretto M, Hariri A R, Sigman M,Bookheimer S Y.: **Neural correlates of facial affect processing in children and adolescents with autism spectrum disorder.** J Am Acad Child Adolesc Psychiatry 2004, **43**:481-490.

111. Wild B, Erb M, Eyb M, Bartels M,Grodd W.: **Why are smiles contagious? An fMRI study of the interaction between perception of facial affect and facial movements.** Psychiatry Res 2003, **123**:17-36.
